# Adjusting the Operational Potential Window as a Tool for Prolonging the Durability of Carbon-Supported Pt-Alloy Nanoparticles as Oxygen Reduction Reaction Electrocatalysts

**DOI:** 10.1021/acscatal.3c06251

**Published:** 2024-03-06

**Authors:** Tina Đukić, Léonard Jean Moriau, Iva Klofutar, Martin Šala, Luka Pavko, Francisco Javier González López, Francisco Ruiz-Zepeda, Andraž Pavlišič, Miha Hotko, Matija Gatalo, Nejc Hodnik

**Affiliations:** † Department of Materials Chemistry, National Institute of Chemistry, Hajdrihova 19, Ljubljana 1001, Slovenia; ‡ Faculty of Chemistry and Chemical Technology, University of Ljubljana, Večna pot 113, Ljubljana 1000, Slovenia; § Department of Analytical Chemistry, National Institute of Chemistry, Hajdrihova 19, Ljubljana 1001, Slovenia; ∥ ReCatalyst d.o.o., Hajdrihova Ulica 19, Ljubljana 1001, Slovenia; ⊥ Department of Catalysis and Chemical Reaction Engineering, National Institute of Chemistry, Hajdrihova 19, Ljubljana 1001, Slovenia; # University of Nova Gorica, Vipavska 13, Nova Gorica 5000, Slovenia

**Keywords:** fuel cells, oxygen reduction reaction, platinum alloys, durability, accelerated degradation tests, potential window, hold time

## Abstract

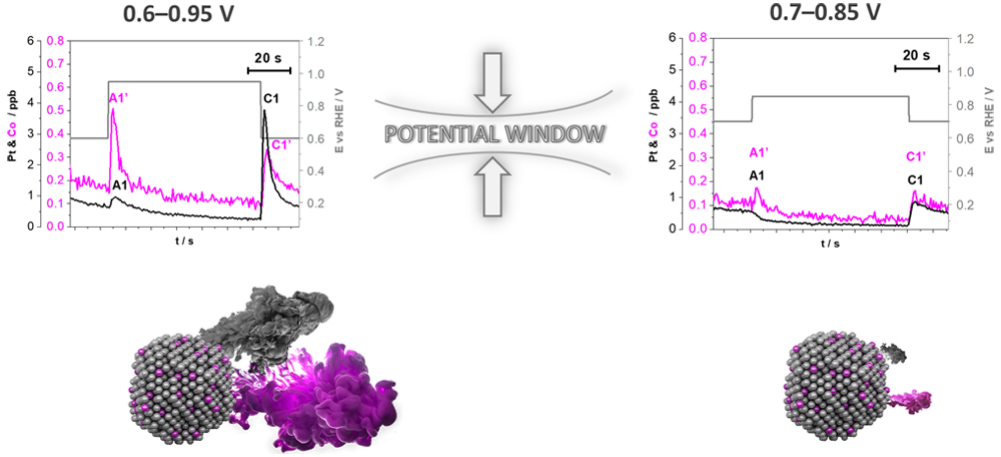

A current trend in the investigation of state-of-the-art Pt-alloys as proton exchange membrane fuel cell (PEMFC) electrocatalysts is to study their long-term stability as a bottleneck for their full commercialization. Although many parameters have been appropriately addressed, there are still certain issues that must be considered. Here, the stability of an experimental Pt-Co/C electrocatalyst is investigated by high-temperature accelerated degradation tests (HT-ADTs) in a high-temperature disk electrode (HT-DE) setup, allowing the imitation of close-to-real operational conditions in terms of temperature (60 °C). Although the US Department of Energy (DoE) protocol has been chosen as the basis of the study (30,000 trapezoidal wave cycling steps between 0.6 and 0.95 V_RHE_ with a 3 s hold time at both the lower potential limit (LPL) and the upper potential limit (UPL)), this works demonstrates that limiting both the LPL and UPL (from 0.6–0.95 to 0.7–0.85 V_RHE_) can dramatically reduce the degradation rate of state-of-the-art Pt-alloy electrocatalysts. This has been additionally confirmed with the use of an electrochemical flow cell coupled to inductively coupled plasma mass spectrometry (EFC-ICP-MS), which enables real-time monitoring of the dissolution mechanisms of Pt and Co. In line with the HT-DE methodology observations, a dramatic decrease in the total dissolution of Pt and Co has once again been observed upon narrowing the potential window to 0.7–0.85 V_RHE_ rather than 0.6–0.95 V_RHE_. Additionally, the effect of the potential hold time at both LPL and UPL on metal dissolution has also been investigated. The findings demonstrate that the dissolution rate of both metals is proportional to the hold time at UPL regardless of the applied potential window, whereas the hold time at the LPL does not appear to be as detrimental to the stability of metals.

## 1 Introduction

Beyond a shadow of a doubt, our generations are witnessing the energy revolution, in which fossil fuels are gradually being replaced by renewable energy sources.^1−3^ In that sense, proton exchange membrane fuel cells (PEMFCs) accompanied by batteries play a key role in establishing fully electrified transportation and replacing conventional internal combustion engine vehicles.^3−5^ To complete the water cycle, PEMFCs will also have to be powered by using “green” hydrogen produced from water using electrolysis where the electrical energy is sourced using emissions-free sources such as wind and solar energy.^6^ However, although establishing supply, storage, and transportation is a grand challenge by itself,^7^ PEMFCs as a “downstream” technology for hydrogen use also have to solve many major bottlenecks to keep up with the massive scalability of hydrogen technologies in the next decade.^8−10^ One of the largest such bottlenecks can be found in the membrane electrode assembly (MEA): the Pt-based electrocatalyst. Although the electrocatalyst is essential for both the anodic hydrogen oxidation reaction (HOR) and the cathodic oxygen reduction reaction (ORR),^9^ the cathode requires many times higher amount of the Pt-based electrocatalyst than the anode resulting from significantly slower kinetics of the ORR.^11^ On the other hand, although there are various very important studies about nonprecious group metal (non-PGM) electrocatalysts,^12−15^ in reality, the only electrocatalyst applied at the production level for PEMFCs used for transport applications is still the Pt-based electrocatalyst.^16,17^ Because of the very high price of deficient Pt, it represents almost half of the total costs of the PEMFC system manufacturing^18^ even when considering the economies of scale.^19^ In addition to other important efforts in the field of Pt-based electrocatalysts (such as core–shell,^20−24^ shape-controlled,^23−26^ as well as nanoframe^23,24,27^ systems), one of the most promising approaches to reduce both the quantity of Pt per vehicle as well as the costs related to platinum per PEMFC system is to alloy Pt with a less noble and at the same time less expensive metal (M = Co, Cu, Fe, or Ni),^16,28−32^ which enables better Pt utilization, i.e., a higher electrochemically active surface area, due to the dilution of Pt atoms inside the nanoparticle (NP) core,^27,33−35^ and a higher activity toward the ORR due to the combination of a ligand, strain, coordination number, and/or surface disorder effects.^36−42^

Nevertheless, although the activity of carbon-supported Pt-based nanoalloys is quite well understood,^4^ the long-term durability of these electrocatalytic systems is still holding many key open questions. Specifically, the fuel cell electrocatalysts are exposed to very aggressive conditions, including a corrosive environment, high temperatures, humidity, frequent stops and starts, fluctuations in the operational voltage, and so forth.^9^ These nonintrinsic operational conditions, together with the intrinsic properties of the electrocatalyst (choice of M,^31^ order/disorder,^30^ dealloying/activation,^43^ type of carbon/degree of graphitization^44^), lead to a spectrum of very complex and interconnected degradation mechanisms: (i) electrochemically induced (transient) dissolution of Pt, which is defined by the thermodynamic tendency of Pt to the formation/reduction of the Pt oxide,^10,45−47^ resulting in Ostwald ripening^48,49^ and/or formation of metallic Pt bands in the membrane^50−52^; (ii) dissolution of M^31,48,53^; and (iii) electrochemical and chemical carbon support corrosion,^54−56^ leading to the agglomeration and/or detachment of whole Pt NPs. These complex instability phenomena are of crucial importance for the longevity of the PEMFCs in the case of their application in both light-duty vehicles (LDVs) and heavy-duty vehicles (HDVs).^57,58^ In particular, as a plan for additional cost reduction, the target for the electrocatalyst loading for LDVs, proposed by the US Department of Energy (DoE) and expected to be achieved by 2025, is less than 0.1 mg_Pt_ cm^–2^.^58^ However, such a low loading of the electrocatalyst can also directly increase its instability and finally significantly affects the overall performance of the fuel cell over time.^59^ Although targeted electrocatalyst loading might not affect the stability of the electrocatalyst in the HDVs to the same degree (0.25 mg_Pt_ cm^–2^),^58^ on the other hand, the average travel distances, and consequently the lifetime requirements of the HDVs, are significantly higher than for the LDVs. In particular, the current goal for longevity of the PEMFCs (expected to be achieved by 2030), proposed by the US DoE, is an operation time of 5000 h for the LDVs (with an ultimate goal of 8000 h, expected to be achieved by 2050) and 25,000 h for the HDVs (with an ultimate goal of 30,000 h, expected to be achieved by 2050), with less than 10% loss in performance.^58,60,61^ Therefore, a comprehensive understanding of the degradation mechanisms of carbon-supported Pt-based nanoalloys is essential. Here, the gap between knowledge obtained at the rotating disk electrode (RDE) and MEA levels must be overcome. For instance, most MEA studies in the literature^45,62^ usually follow the US DoE degradation protocol for fuel cells and fuel cell components.^58^ The protocol consists of 30,000 trapezoidal wave cycles in a voltage range similar to that which is expected for an automotive drive cycle (0.6–0.95 V), with a 3 s hold at each potential, at 80 °C, thus simulating close-to-real operational conditions regarding the operational lifetime of 5000 h as the current target for LDVs.^58,63^ On the other hand, apart from rare studies such as the recent one presented by Imhof et al.,^64^ RDE stability studies typically vary with respect to this protocol.

Furthermore, the dependence of the stability of carbon-supported Pt-based nanoalloys not only on intrinsic but also on nonintrinsic factors should be addressed in both RDE and MEA. For instance, in addition to the effect of temperature,^48,53,54,65^ the impact of the potential window on the stability of carbon-supported Pt-based nanoalloy electrocatalysts has also recently been shown and extensively described. Namely, it has been clear for some time that an increase in upper potential limit (UPL) strongly affects the stability of carbon-supported Pt-nanoalloys, in terms of both carbon support corrosion^54^ and metal dissolution.^31,46,66−69^ Moreover, in terms of metal dissolution, in our recent work,^48^ it has been shown that at a constant high temperature (75 °C), the general widening of the potential window linearly increases the degradation rate of Pt-Co NPs in the following order: 0.7–0.925 V_RHE_ < 0.7–1.0 V_RHE_ < 0.6–0.925 V_RHE_ < 0.6–1.0 V_RHE_ < 0.4–1.0 V_RHE_. Further widening of the potential window (0.4–1.2 V_RHE_), however, results in exponential growth of the degradation rate. An additional explanation has been provided in another one of our previous studies^43^ where it has been clearly shown that, in addition to the UPL,^68^ the lower potential limit (LPL) also strongly affects the metal dissolution of the state-of-the-art carbon-supported Pt-based nanoalloys. Namely, varying the LPL in the range of 0.7/0.65/0.6 V_RHE_, with a constant UPL (0.925 V_RHE_), provided evidence that the cathodic dissolution of both Pt as well as less noble metal increases with a decrease in LPL. In line with this study, Ronovsky et al.^45^ provided a comprehensive XRD study on the stability of an octahedral-PtNiIr electrocatalyst during MEA square-wave accelerated degradation tests (ADTs) with a constant UPL (0.95 V) and LPL varying from 0.6 to 0.7 V. Dissolution of Pt and Ni has been tracked by observing three XRD parameters (scattering intensity, lattice parameter, and crystallite size), and a strong dependence of the stability of Pt-alloy based electrocatalyst on the LPL has been confirmed once again. The authors have observed that the extent of degradation of the analyzed electrocatalyst is proportional not only to the degree of Pt oxidation but also to the degree of reduction of the formed Pt oxide. Furthermore, an extensive study of the operated Toyota Mirai stack, provided by Argonne National Laboratory, showed that keeping the operational potential window in the range of 0.65–0.85 V_RHE_ instead of the 0.6–0.95 V_RHE_ specified by the DoE does not lead to Co dissolution into the membrane,^70^ which is crucial for PEMFC longevity.^19,51,52,71−75^ All of these findings serve as a good indicator that adjusting the potential/voltage window (in a half-cell/MEA, respectively) can have severe implications on the long-term stability of Pt-alloy electrocatalysts.

As a part of this work, the stability of an experimental dealloyed Pt-Co/C electrocatalyst from ReCatalyst has been comprehensively investigated. The paper has two goals: (1) to provide the stability guidelines to the PEMFC scientific community for testing of Pt-based electrocatalysts in liquid half-cells using RDE, which are based on the *Degradation protocol for fuel cells and fuel cell components* proposed by the US DoE, and (2) to provide novel insights into the significant effects of both the LPL and UPL limits on the dissolution of metals (Pt and M) to understand also how to minimize it during PEMFC operation as much as possible. To achieve these goals, two previously reported advanced electrochemical methodologies have been used: (i) a high-temperature disk electrode (HT-DE) setup, which enables ADT to be performed in a liquid electrolyte half-cell by utilization of a standard RDE at elevated temperatures, and (ii) an electrochemical flow cell coupled to an inductively coupled plasma mass spectrometer (EFC-ICP-MS), which allows for precise, highly sensitive (ppb range) time-temperature-and-potential resolved measurements of dissolution of metals, as well as direct monitoring of the dissolution mechanisms at close-to-real operational conditions.^10,31,48,66,67,76−80^

## 2 Experimental Section

### 2.1 Synthesis of the Experimental Dealloyed Pt-Co/C Electrocatalyst

The model and experimental dealloyed Pt-Co/C electrocatalyst by ReCatalyst was prepared following the processes already reported previously.^81,82^ Briefly, the electrocatalyst was prepared in three steps. In the first step, Pt NPs were deposited onto a commercial carbon black support (Ketjen Black EC300J) via the double passivation galvanic displacement method reported elsewhere.^81^ In the second step, the prepared composite with deposited Pt NPs was thermally annealed to obtain a Pt-alloy crystal phase. In the last step, dealloying (acid washing) was performed in accordance with the work described previously.^83−85^ The final electrocatalyst resulted in a composition with 28.6 wt % Pt and 1.4 wt % Co.

### 2.2 XRD Analysis

The powder X-ray diffraction (XRD) measurements of samples containing Co were carried out on a PANalytical X’Pert PRO diffractometer with Cu Kα radiation (λ = 1.541874 Å) in the 2θ range from 10 to 60° with a 0.039° step per 300 s using a fully opened Pixcel detector. Samples were prepared on zero-background Si holders.

### 2.3 Transmission Electron Microscopy (TEM) Analysis

Scanning transmission electron microscopy (STEM) high-angle annular dark field (HAADF) and bright field (BF) imaging was carried out in a probe Cs-corrected scanning transmission electron microscope (Jeol ARM 200 CF) operated at 80 kV. Samples were prepared on a holey carbon Cu grid.

### 2.4 Energy-Dispersive X-ray (EDX) Analysis

Energy-dispersive X-ray (EDX) analysis was performed using a detector (SDD Ultim max 100, Oxford, UK) at 20 kV. Samples were prepared using the following procedure: A small amount of powder electrocatalyst sample (1–3 mg) was put on a 13 mm polished metal disk and covered with a metal disk of the same size. Samples were pelleted with a manual press until a pellet with a thickness of about 50 μm was obtained. Standard scanning electron microscope (SEM) pin mounts (Agar Scientific) covered with conductive carbon tape (Agar Scientific) were used to hold the pelleted samples.

### 2.5 Accelerated Degradation Tests Using the High-Temperature Disk-Electrode (HT-DE) Methodology

#### 2.5.1 High-Temperature Disk Electrode (HT-DE) Setup

The accelerated degradation tests (ADTs) were performed in a setup already described as a part of our previous work.^10,48,54^ Briefly, the setup was composed of a two-compartment HT cell using 0.1 M HClO_4_ electrolyte (Carl Roth, Rotipuran Supra) with a conventional three-electrode system controlled by a potentiostat (SP-200 Potentiostat, Biologic). A reversible hydrogen electrode (HydroFlex) was used as a reference (separated from the working electrode in a different compartment via a salt bridge), and a graphite rod was used as a counter electrode. To prevent evaporation of the electrolyte due to prolonged ADTs at high temperature (60 °C), in addition to the condenser, the other open parts of the HT-DE cell (the capillary for the counter electrode, the capillary for the salt bridge, and the beaker for the reference electrode) were covered by the in-house designed PEEK caps.

#### 2.5.2 Thin-Film Rotating Disk Electrode (TF-RDE) Setup

Whereas the ADTs were performed in the HT-DE setup, oxygen reduction reaction (ORR) polarization curves and CO electrooxidation cyclovoltammograms (CVs) both before as well as after the ADT were measured in a typical TF-RDE setup also in accordance to our previous work.^10,48,54^ Electrochemical measurements were conducted with an SP-200 Potentiostat (Biologic) in a two-compartment electrochemical cell in a 0.1 M HClO_4_ electrolyte with a conventional three-electrode system. Similarly, a reversible hydrogen electrode (HydroFlex) was used as a reference electrode, and a graphite rod was used as a counter electrode.

#### 2.5.3 Preparation of the Setups and the Thin Films

Extensive cleaning was performed to eliminate any organic and inorganic impurity contributions that could potentially affect the stability of the studied electrocatalysts. Prior to the set of degradation experiments, all of the glassware was soaked in both a base bath (mixture of KOH and isopropanol) and an acid bath (mixture of conc. HNO_3_ and H_2_SO_4_) as well as boiled in distilled water three times. Before each experiment, the HT cell was heated for 2 h at 90 °C in 0.1 M HClO_4_ and then boiled in Milli-Q water for 2 h, whereas the RT cell was boiled in distilled water for 1 h.

The working electrode was a glassy carbon (GC) disk embedded in Teflon (Pine Instruments) with a geometric surface area of 0.196 cm^2^. The GC electrode was polished to a mirror finish with Al_2_O_3_ paste (particle size 0.05 μm, Buehler) on a polishing cloth (Buehler). After polishing, the electrode was rinsed and ultrasonicated (Ultrasonic cleaner, ASonic) first in a Milli-Q/isopropanol mixture and then only in Milli-Q several times for 5 min. Once the GC electrode was prepared, 20 μL of 1 mg mL^–1^ freshly prepared water-based well-dispersed electrocatalyst ink was pipetted on the electrode completely covering it and dried under ambient conditions. Such preparation resulted in an electrocatalyst loading of 20 μg, i.e., 0.1 mg cm^–2^, for all electrocatalysts. After the drop had dried, 5 μL of Nafion solution (ElectroChem, 5% aqueous solution) diluted in isopropanol (1:50) was added. The electrode was then mounted on a rotator (Pine Instruments).

#### 2.5.4 Electrochemical Characterization

The electrode was then initially placed in the TF-RDE setup in an inert gas-saturated electrolyte (0.1 M HClO_4_) under potential control at 0.05 V_RHE_ using a rotator (Pine Technologies). All electrocatalysts were electrochemically activated (50 cycles between 0.05 and 1.2 V_RHE_ with a scan rate of 300 mV s^–1^ under a rotation rate of 600 rpm). After the activation, the electrolyte was exchanged for a fresh one. ORR polarization curves were measured in an oxygen-saturated electrolyte with rotation at 1600 rpm in the potential window 0.05–1.0 V_RHE_ with a scan rate of 20 mV s^–1^. At the end of the ORR polarization curve measurement, the electrolyte was purged with CO under a potentiostatic mode (0.05 V_RHE_) to ensure successful CO adsorption. Afterward, the remaining CO in the electrolyte was displaced, and the electrolyte was saturated with N_2_. CO electrooxidation was performed using the same potential window and scan rate as in ORR, but without rotation and in an N_2_ saturated electrolyte. The electrochemically active surface area (ECSA_CO_) was determined by integrating the charge in CO electrooxidation (“stripping”) experiments as described in ref (86). For the ORR, after subtraction of background current (due to capacitive currents), kinetic parameters, i.e., specific activity (SA) and mass activity (MA), were calculated at 0.95 V_RHE_. The ohmic resistance of the electrolyte was determined and compensated for as reported in ref (87). Afterward, the working electrode was carefully transferred to the HT-DE setup (taking care to not introduce any impurities during the transfer process), and an ADT was performed composed of trapezoidal wave cycling (various numbers of cycles were applied: 1000/5000/10,000/30,000) at 60 °C 0.1 M HClO_4_ and at various potential windows (LPL–UPL; LPL = 0.6/0.7 V_RHE_ and UPL = 0.8/0.95 V_RHE_, where LPL is the lower potential limit and UPL is the upper potential limit; 0.7 V s^–1^, 3 s hold at both LPL and UPL). After the last ADT cycle, the working electrode was again carefully transferred back to the standard TF-RDE setup, and the ORR polarization curve as well as CO electrooxidation was measured once again (at RT). This approach allowed interpretation of the results in terms of loss of ECSA_CO_ and loss of kinetic parameters (SA and MA), calculated at a fixed potential. However, it is worth mentioning that other methods can also be used to describe the kinetic properties of aged electrocatalysts.^88^

#### 2.5.5 *Ex Situ* ICP-MS for the Determination of Metals in the Electrolyte after ADTs

*Ex situ* samples for determination of metal concentrations were collected after the ADTs and analyzed by using ICP-MS. Samples were diluted 10 times prior to measurement. For the preparation of standards, ultrapure water (Milli-Q, Millipore) and ultrapure acid (HClO_4_; Carl Roth, Rotipuran Supra) were used. Standards were prepared in-house by dilution of certified, traceable, inductively coupled plasma (ICP)-grade single-element standards (Merck Certipur). An Agilent quadrupole ICP-MS instrument (Agilent 7900, Agilent Technologies, Santa Clara, CA) equipped with a MicroMist glass concentric nebulizer and Peltier-cooled, Scott-type spray chamber was used for the measurements. Each *ex situ* electrolyte sample was measured three times, and RSD for each measurement was determined. The typical RSD for Co was 3%, whereas the amount of dissolved Pt was too low for accurate and relevant *ex situ* determination.

### 2.6 Electrochemical Flow Cell Coupled to Inductively Coupled Plasma Mass Spectrometry (EFC-ICP-MS)

#### 2.6.1 Electrochemical Flow Cell (EFC)

The setup and measurement guidelines have been established as part of the previous work.^10,31,48,66,67,76−80^ Briefly, the working and counter electrodes in the electrochemical flow cell (EFC) were glassy carbon disks (3 mm diameter) embedded into the PEEK material (BASi). The disks were aligned in series; the counter electrode was placed first, and the working electrode was placed second in the direction of the electrolyte flow. The sample was deposited on the electrode by drop casting a 5 μL drop of the ultrasonically homogenized electrocatalyst ink (1 mg mL^–1^). Such preparation resulted in an electrocatalyst loading of 5 μg, i.e., 0.07 mg cm^–2^, for all electrocatalysts. In addition, to increase the surface area of the counter electrode, a 5 μL drop of Ketjen Black EC300J suspension (1 mg mL^–1^) was deposited on the glassy carbon counter electrode. After the drop had dried, 5 μL of Nafion solution (ElectroChem, 5% aqueous solution) diluted in isopropanol (1:50) was added, covering both electrodes at the same time. The Ag|AgCl reference electrode potential against RHE was determined before the start of the experiment. The housing of the cell was made from the PEEK material, and the design was modeled after a commercial cross-flow cell (BASi, MF-1092, cross-flow cell). The volume of the cell was established with a homemade silicon gasket with 0.3 mm thickness and 1.5 cm^2^ ellipsoidal cut. The electrolyte (0.1 M HClO_4_) was pumped through the cell at a constant flow of 400 μL min^–1^. Two syringes using a Luer Lock connection to a polytetrafluoroethylene (PTFE) tubing, two syringe pumps (WPI AL1000-220Z), and a diagonal four-way flow valve (Idex, V-100D) were used to enable a continuous flow of the solution. Additionally, to avoid possible memory effects, each measurement protocol was performed on a fresh electrocatalyst film. Also, each experiment was performed at least two times for reproducibility.

#### 2.6.2 ICP-MS

The EFC was coupled with an ICP-MS instrument (Agilent 7900ce; Agilent Technologies, Palo Alto, CA) equipped with a MicroMist glass concentric nebulizer and a Peltier-cooled, Scott-type double-pass quartz spray chamber. The signals were recorded for Co^59^ and Pt^195^ with 0.5 s integration per data point. To convert the ICP-MS signals to concentration (ppb), standard solution of Co and Pt in 0.1 M HClO_4_ was recorded with the following concentrations: 1, 2, 5, 10, 20, and 50 ppb.

#### 2.6.3 Electrochemical Protocol

**Scheme 1:**
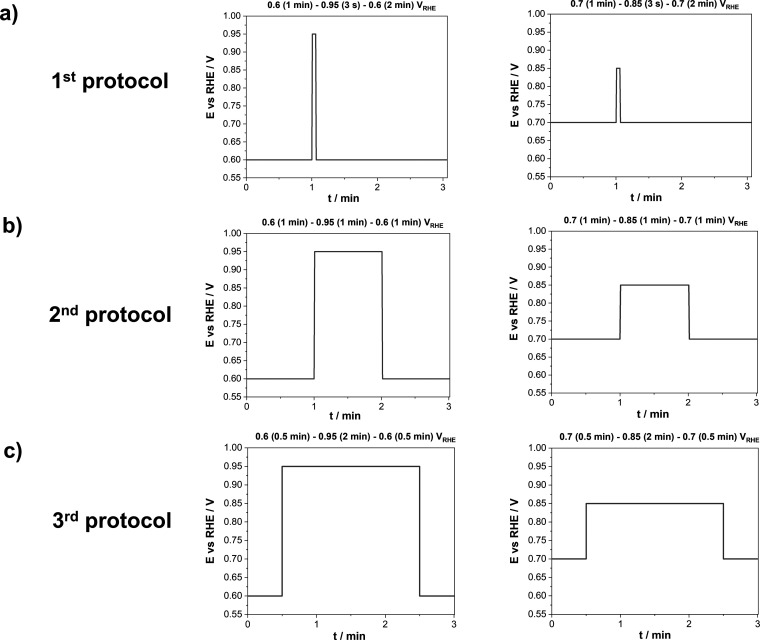
Profiles of Potential Protocols Corresponding to a Single Cycle of ADT Performed in the EFC-ICP-MS Setup by Trapezoidal Wave Cycling

Electrochemical experiments were performed with an SP-200 Potentiostat (Biologic) with a typical three-electrode setup. No ohmic drop compensation method was used. Initially, Milli-Q water was pumped through the cell under open circuit conditions (OCP) before switching to 0.1 M HClO_4_. After a steady background had been reached (for at least 2 min), the potentiodynamic protocol was started; to check for the effect of the potential window and the hold time at each potential, the electrocatalysts were cycled for trapezoidal wave cycles between LPL and UPL with various hold times at each potential, with 10 cycles in total and an adequate scan rate to go from one potential limit to the next in 0.5 s to avoid fast potential jumps (0.7 V s^–1^ for 0.6–0.95 V_RHE_ and 0.3 V s^–1^ for 0.7–0.85 V_RHE_; (see Scheme 1 and Table 1): (1) LPL = 0.6/0.7 V_RHE_ (1 min hold) → UPL = 0.95/0.85 V_RHE_ (3 s hold) **→** LPL = 0.6/0.7 V_RHE_ (2 min hold) (depicted as the first protocol at Scheme 1a); (2) LPL = 0.6/0.7 V_RHE_ (1 min hold) → UPL = 0.95/0.85 V_RHE_ (1 min hold) **→** LPL = 0.6/0.7 V_RHE_ (1 min hold) (depicted as the second protocol at Scheme 1b); and (3) LPL = 0.6/0.7 V_RHE_ (0.5 min hold) → UPL = 0.95/0.85 V_RHE_ (2 min hold) → LPL = 0.6/0.7 V_RHE_ (0.5 min hold) (depicted as the third protocol at Scheme 1c). Furthermore, after each experiment, a sequence of potential pulses was performed to synchronize the electrochemical experiment with the ICP-MS signal.

**Table 1:** EFC-ICP-MS Measurement Protocols

	**(i)**	**(ii)**	**(iii)**
1^st^ protocol	1 min hold at LPL (0.6 or 0.7 V_RHE_)	3 s hold at UPL (0.95 or 0.85 V_RHE_)	2 min hold at LPL (0.6 or 0.7 V_RHE_)
2^nd^ protocol	1 min hold at LPL (0.6 or 0.7 V_RHE_)	1 min hold at UPL (0.95 or 0.85 V_RHE_)	1 min hold at LPL (0.6 or 0.7 V_RHE_)
3^rd^ protocol	0.5 min hold at LPL (0.6 or 0.7 V_RHE_)	2 min hold at UPL (0.95 or 0.85 V_RHE_)	0.5 min hold at LPL (0.6 or 0.7 V_RHE_)

## 3 Results and Discussion

**Figure 1:**
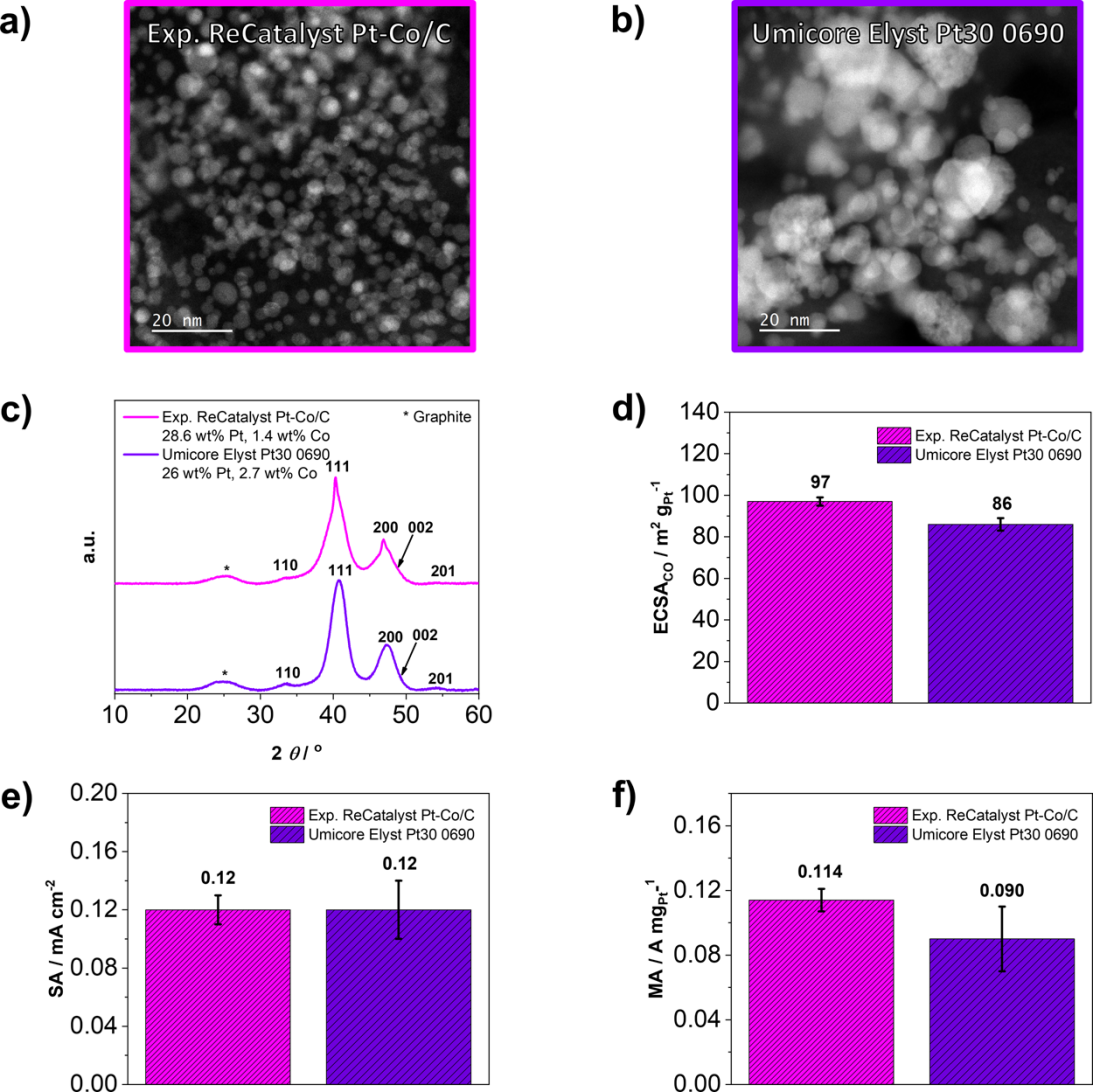
(a, b) HAADF STEM, (c) XRD, and (d–f) TF-RDE comparison between experimental ReCatalyst Pt-Co/C and Umicore Elyst Pt30 0690 Pt-Co/C benchmark. Additional characterization is available in SI Figures S1–S4 and Table S1. In all figures, magenta is used for the data corresponding to the experimental ReCatalyst Pt-Co/C, whereas violet is used for the data corresponding to the Umicore Elyst Pt30 0690 Pt-Co/C benchmark.

For this study, an experimental and model Pt-Co/C electrocatalyst from ReCatalyst has been investigated. To confirm the viability of the model electrocatalyst, a comparison between the model Pt-Co/C electrocatalyst from ReCatalyst and a commercially available Pt–Co/C benchmark from Umicore (Elyst Pt30 0690) is presented in Figure 1. Based on the TEM analysis (Figure 1a,b and Figures S1 and S2) and the corresponding particle size distributions (Figure S3), it is evident that the Umicore benchmark has a slightly larger average particle size than the experimental electrocatalyst from ReCatalyst. Figure 1c depicts a comparison of the XRD analysis of both respective electrocatalysts where a slight difference in the position of the main 111 and 200 peaks is visible toward lower angles for the experimental Pt-Co/C electrocatalyst from ReCatalyst. As indicated in the same figure, this is well in line with a slightly more Pt-rich composition of the ReCatalyst electrocatalyst (28.6 wt % Pt and 1.4 wt % Co) in comparison with the Umicore benchmark (26 wt % Pt and 2.7 wt % Co) as determined by the EDX-SEM methodology (see also SI Table S1). A comparison of the initial electrochemical properties obtained by TF-RDE analysis is presented in Figure 1d–f. Because the Umicore benchmark has a larger particle size compared to the experimental ReCatalyst Pt-Co/C, one would also expect a significantly lower ECSA_CO_ in the case of the Umicore benchmark. However, the experimental ReCatalyst Pt-Co/C possesses an ECSA_CO_ that is only slightly higher than that of the Umicore benchmark, which can be attributed to the difference in structures. Namely, whereas the Pt-Co/C from ReCatalyst has a typical core–shell structure, the Pt-Co/C benchmark from Umicore also has a visible fraction of larger and so-called “spongy” particles. This porous structure, usually formed during dealloying process, additionally boosts the surface area,^89,90^ which could be the reason why Umicore’s Pt-Co/C has a more similar ECSA_CO_ to the experimental Pt-Co/C from ReCatalyst than expected. Furthermore, the two are rather similar in terms of SA, which was expected. As a consequence of slightly higher ECSA_CO_,^61^ the MA of experimental ReCatalyst Pt-Co/C is also higher in comparison to the MA of the Umicore benchmark.

The stability of the dealloyed Pt-Co/C electrocatalyst has been investigated by the voltage trapezoidal wave *Degradation protocol for fuel cells and fuel cell components* proposed by the US DoE. The original DoE protocol for the ADT consists of 30,000 trapezoidal wave potential steps between 0.6 and 0.95 V with a 3 s hold time at both LPL and UPL and rise time of ∼0.5 s or less at 80 °C.^58^ The goal of such a protocol is to imitate realistic conditions of a PEMFC for LDVs^58,63^ as much as possible regarding the target operational lifetime, the potential range of an automotive drive cycle, as well as the waiting time due to traffic conditions (e.g., traffic lights, traffic jams).^91^ Furthermore, when it comes to the LDVs, it refers to the operational potential range where the metal dissolution is expected to be dominant^47,59,91,92^; especially during trapezoidal wave cycling with potential holds, the impact of the carbon support corrosion on the electrocatalyst performance is supposed to be minimized but still present.^62^ However, it is important to emphasize that these tests are intended for the stability tests in the solid-electrolyte MEA,^58^ whereas in this work, the ADTs have been performed in a modified liquid-electrolyte high-temperature half-cell setup. Therefore, the DoE ADT protocol was slightly modified and adapted for this research. For instance, although the proposed temperature for the ADT is 80 °C, herein, 60 °C has been used to minimize the evaporation of the liquid electrolyte (0.1 M HClO_4_) and thus be far enough from the boiling point of water during such long-lasting experiments (>60 h for 30,000 cycles). Furthermore, as part of our prior research,^48,54^ we have already shown that, in contrast to room temperature, the effect of temperature already has a significant impact at temperatures above 50 °C. Thus, a balanced approach of considering the properties of water (namely, minimizing evaporation) as well as having a large enough impact of temperature on the durability has been chosen as the most sensible approach. Lastly, the usage of liquid half-cells also limits one to follow the guidelines related to the relative humidity, flow of reactants, etc., which are also relevant parameters when performing the DoE ADT protocol in an MEA.^58^ Furthermore, whereas the DoE protocol proposes periodic measurements of the electrochemical metrics during ADT, here ECSA_CO_, SA, and MA have been evaluated only before and after each ADT experiment (*ex situ*). In other words, a new experiment has been performed for 1000, 5000, 10,000, and 30,000 ADT cycles. This is because after every measurement, the electrolyte has been collected for the *ex situ* ICP-MS analysis and determination of the dissolved Co. Prior research has also revealed that too frequent periodic measurements during the ADTs result in additional degradation of the Pt-alloy cathodes, thus influencing the overall results.^93^ Given that the ADTs proposed by the DoE are rather time and energy consuming as well as extremely slow in terms of the throughput even when using our modified liquid high-temperature half-cell setup, the initial DoE evaluation in this study has also served to determine the optimal length of further in-depth analysis. In other words, an additional goal of this study was also to determine the experimental conditions suitable for optimal throughput and data generation, which provide relevant information about the degradation of Pt-based electrocatalysts.

**Figure 2:**
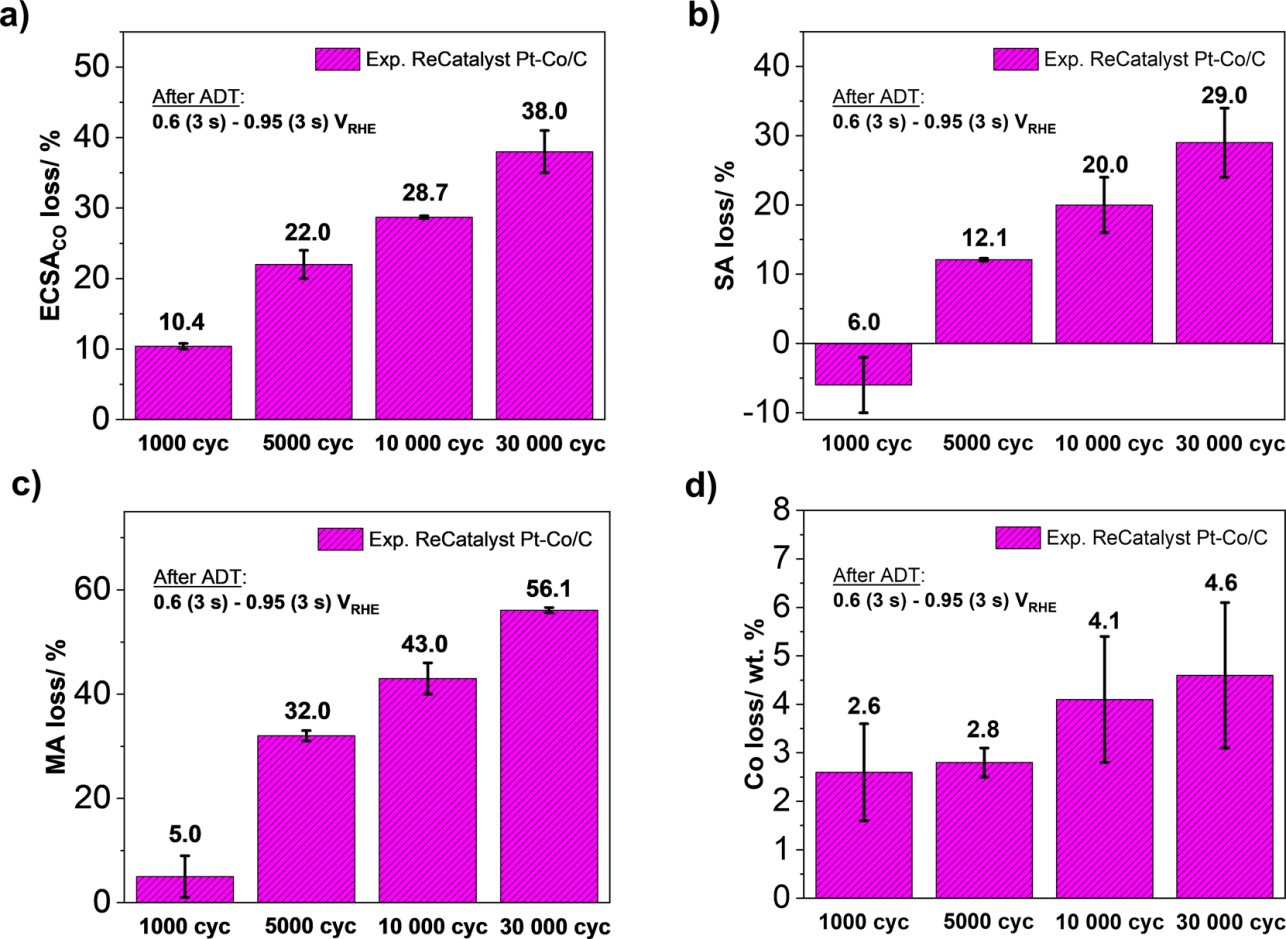
Comparison of the different number of cycles (1000, 5000, 10,000, and 30,000) of accelerated degradation tests (ADTs) performed at a constant potential window with a 3 s hold at both LPL and UPL (0.6 (3 s)–0.95 (3 s) V_RHE_, with a 0.5 s rise time between each potential, 0.7 V s^–1^, 0.1 M HClO_4_, 60 °C): (a) ECSA_CO_ loss, (b) SA loss, (c) MA loss, and (d) Co loss of the ReCatalyst dealloyed Pt-Co/C electrocatalyst.

Figure 2a shows the loss of ECSA_CO_ (%) after ADT consisting of 1000, 5000, 10,000, or 30,000 cycles at a constant potential window with a 3 s hold at both LPL and UPL (0.6 (3 s)–0.95 (3 s) V_RHE_, with a 0.5 s rise time between each potential at a scan rate of 0.7 V s^–1^, 0.1 M HClO_4_, 60 °C) performed in our in-house designed high-temperature HT-DE setup. The following trend of ECSA_CO_ loss has been observed in line with the expectations: 1000 cycles < 5000 cycles < 10,000 cycles < 30,000 cycles. A similar trend has also been observed for SA loss (Figure 2b) as well as MA loss (Figure 2c). Interestingly, however, an increase in the measured SA after 1000 ADT cycles has been observed rather than a decrease as in the case of other measurements of 5000 cycles or more. We presume that in these initial ADT cycles, the electrocatalyst underwent further electrochemical activation (e.g., additional removal of Co from the topmost surface layers as shown in Figure 2d, but also other phenomenon such as reshaping of the particles etc.^66,80^). On the other hand, in accordance with the expectations, a slight decrease in the SA is observed after 5000 cycles of ADT, indicating that the degradation has reached the point of decay in the ligand and/or strain effects.^52,94^ Additional cycling (up to 10,000 and 30,000 cycles) leads to further degradation of the electrocatalyst and a further linear increase in the SA and MA loss (Figure 2b,c). According to the obtained results from Figure 2a–d (see also SI Figures S5 and S6), it can be concluded that there is some point of limit between activation and degradation because both processes are based on similar principles, e.g., dealloying of the less noble metal (in this case, Co) from the Pt-NPs. Therefore, it is critical to ensure that an adequate number of the ADT cycles (while of course also using an adequate ADT protocol) are performed to efficiently study the degradation of Pt-based electrocatalysts. Nevertheless, although we can assume that a significant rate of degradation has already been achieved after 5000 cycles, the results show that further cycling will give rise to a linear increase in degradation. On the other hand, taking into account the difference in the length of ADTs (approximately 20 h for 10,000 cycles and 60 h for 30,000 cycles) and by the results, we can conclude that we already gain sufficient data on the degradation of an electrocatalyst after 10,000 cycles under the specified ADT protocol conditions. Consequently, to establish a compromise between valuable information on ADTs and time requirements, we have determined that the RDE-level stability tests can be carried out by performing 10,000 cycles of ADT instead of the 30,000 cycles proposed by the DoE. Thus, for this research, all further ADTs are performed by running 10,000 cycles of ADT.

**Figure 3:**
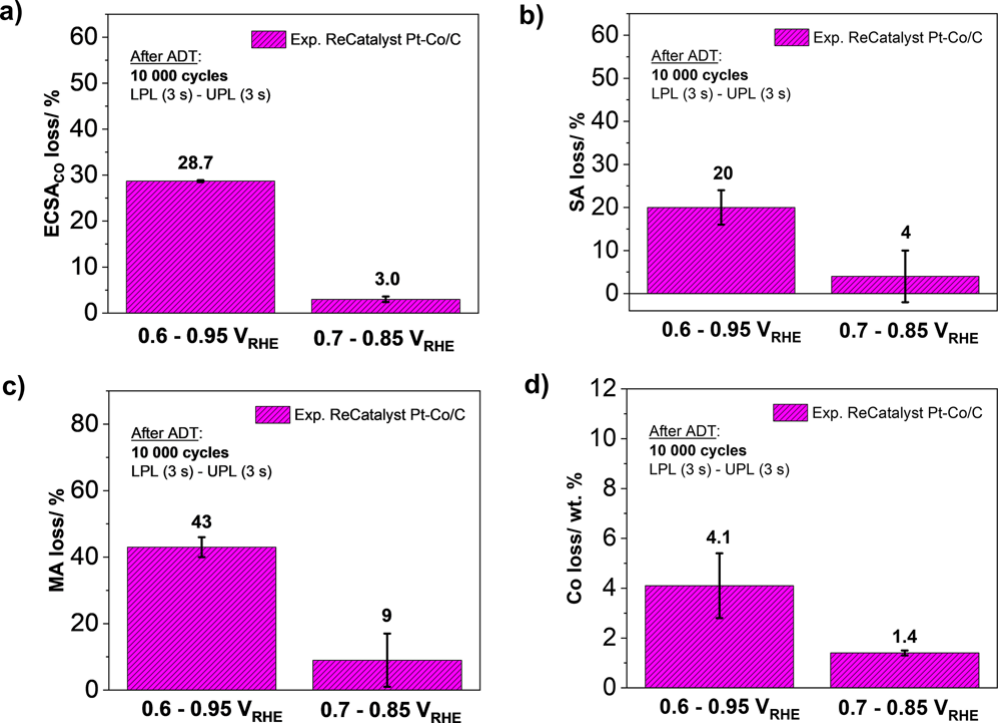
Effect of varying the operational potential window on accelerated degradation tests (ADTs) (LPL (3 s)–UPL (3 s); LPL = 0.6/0.7 V_RHE_ and UPL = 0.85/0.95 V_RHE_; 10,000 cycles, 0.7 V s^–1^, 0.1 M HClO_4_, 60 °C): (a) ECSA_CO_ loss, (b) SA loss, (c) MA loss, and (d) Co loss of the ReCatalyst dealloyed Pt-Co/C electrocatalyst demonstrated using the HT-DE setup.

In continuation, we have tested the hypothesis based on our prior work^43,48^ that further narrowing of the potential window below 0.6–0.95 V will have a severe impact on the degradation of the Pt-alloy cathodes. Figure 3 represents a comparison of performing the same 10,000 cycles of the trapezoidal wave high-temperature ADTs but changing (narrowing) the potential window. More precisely, the LPL has been varied from 0.6 to 0.7 V_RHE_, whereas the UPL has been varied from 0.95 to 0.85 V_RHE_. In accordance to the expectations, clear evidence of a dramatically decreased loss of ECSA_CO_ (Figure 3a) and SA (Figure 3b) and, correspondingly, MA loss (Figure 3c) as well as Co loss (Figure 3d) has been observed. This significant decrease in ECSA loss can be explained by the lower Pt dissolution and the consequent Ostwald ripening.^10,65^ Additionally, lower Co dissolution, which is already known to always follow Pt dissolution (just like other 3d transition metals),^10,31,43,48,66^ results in the substantially lower loss of SA because it is very well-known that the SA of Pt-nanoalloys is dependent on the ligand, strain, coordination number, and surface disorder effects provided by the presence of less noble metal.^37−42,95^ Thus, narrowing the potential window to only 0.7–0.85 V_RHE_ has a dramatic effect on the durability of Pt-alloy NPs, drastically decreasing the loss of ECSA_CO_ and SA (and consequently MA loss) due to the decreased dissolution of metals.

To gain additional mechanistic insights and understand the influence of the trapezoidal wave degradation ADT protocols on the dissolution of metals, an online analysis of metal dissolution has been performed using the previously already widely described EFC-ICP-MS methodology.^10,31,43,48,66,77,96,97^ However, to ensure adequate resolution between the cathodic and anodic Pt and Co dissolution peaks, we adjusted the protocol by prolonging the potential hold periods (Scheme 1 in the Experimental Section). Specifically, three trapezoidal wave protocols of 10 cycles each were performed in accordance with Table 1 (see the Experimental Section). This number of cycles has been sufficient to observe a trend in dissolution about the applied potential window. The most important is, however, that for both the potential window proposed by DoE (0.6–0.95 V) as well as the narrower potential window (0.7–0.85 V), different UPL potential holds have been investigated to enable dissolution mechanism interpretation, highly relevant for Pt-based NPs, as well as to evaluate the impact of both parameters (potential window and hold time at the UPL) on the amount of Pt and Co dissolution. Furthermore, the number of cycles as well as duration of one cycle was approximately equal for each protocol (ca. 3 min per cycle, 10 cycles in total); thus, the total time of each experiment was the same (ca. 30 min), enabling one to exclude the effect of the number of cycles^69^ on the metal dissolution.

**Figure 4:**
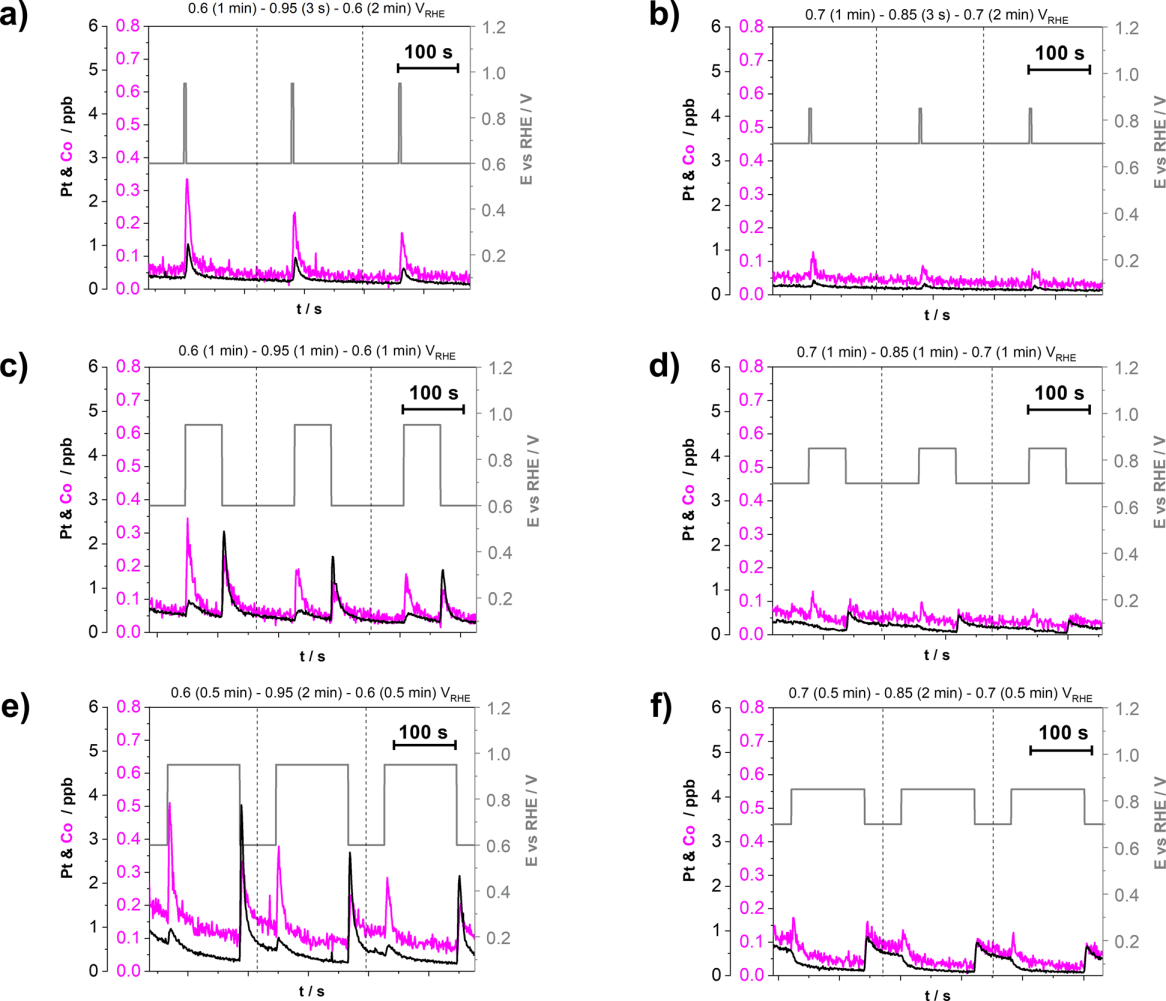
Effect of potential window (0.6–0.95 and 0.7–0.85 V_RHE_) and different hold times at both LPL and UPL on the quantity of metal dissolution (Pt and Co) during trapezoidal wave cycling between LPL and UPL (0.7 and 0.3 V s^–1^, respectively, 10 cycles in total) demonstrated using the EFC-ICP-MS setup in the flow of 0.1 M HClO_4_. Three different protocols per cycle were used: (a, b) LPL (1 min)–UPL (3 s)–LPL (2 min), (c, d) LPL (1 min)–UPL (1 min)–LPL (1 min), and (e, f) LPL (0.5 min)–UPL (2 min)–LPL (0.5 min). For better resolution, close-up metal dissolution profiles of the fourth, fifth, and sixth cycles, where the dissolution rate approached steady-state value and there is no effect of a fresh electrocatalyst film, are presented. Each metal has its own *Y* axis to better compare the profiles despite the detected concentration differences. The gray lines represent the cycles between LPL and UPL. The transition between different cycles is denoted by dashed lines. Supplementary experiments during cycling between LPL (3 s) and UPL (3 s) are provided in SI Figure S10. The entire metal dissolution profiles of a total of 10 cycles for each protocol for both experimental ReCatalyst Pt-Co/C and benchmark from Umicore (Elyst Pt30 0690) are also available in SI Figures S11 and S12, respectively.

**Figure 5:**
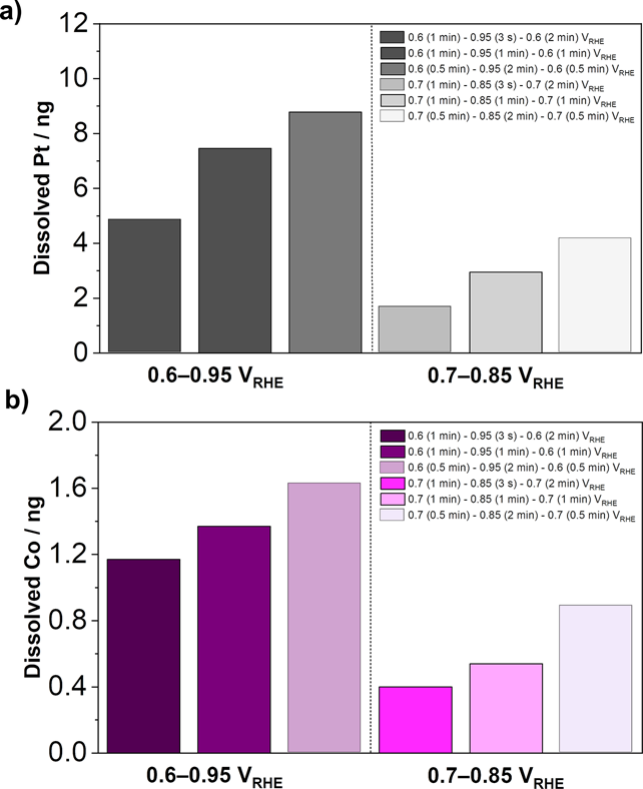
Amount of dissolved (a) Pt and (b) Co obtained by integrating corresponding peaks from dissolution profiles of Pt and Co during 10 trapezoidal wave cycles between LPL = 0.6/0.7 V_RHE_ and UPL = 0.85/0.95 V_RHE_, demonstrated using the EFC-ICP-MS setup (see Figure S11 and Table S2).

Figure 4 shows the simultaneous effect of varying the operational potential window and the hold time (at both LPL and UPL) on the dissolution of Pt and less noble metal from the dealloyed Pt-Co/C electrocatalyst. To ensure better visibility of the metal dissolution peaks, only three cycles are presented, namely, the fourth, fifth, and sixth cycle (see SI Figures S11 and S12 for the full experiments). Information about the amount of dissolved metals during all 10 cycles for each protocol is summarized in Figure 5.

Focusing on the varying hold times at both LPL and UPL (Figure 4a,c,e and b,d,f), whereas the hold time at LPL does not have an important impact on the overall dissolution of both Pt and Co, interestingly, the hold time at UPL significantly affects the quantity of dissolution of both metals. In other words, a longer hold at UPL increases Pt and Co dissolution in the following order: 3 s < 1 min < 2 min. In particular, the total amount of dissolved Pt and Co for both potential windows (0.6–0.95 and 0.7–0.85 V_RHE_) increases with increasing the hold time at UPL. This is in line with the results from Kneer et al.^98^ as well as the observation from Della Bella et al.,^69^ which have shown in the MEA setup that not only a higher UPL but also a longer hold time at the UPL results in a higher ECSA loss^98^ or a higher decrease of the cathode’s roughness factor due to an increase in Pt dissolution/redeposition, resulting in faster H_2_/air performance degradation.^69^ What is worth mentioning here is that the effect of number of cycles, which is also shown to have a great impact to the stability of Pt NPs,^69^ is excluded in the present study. Therefore, the hold time at UPL mostly affects Pt and Co dissolution per cycle while revealing the dissolution mechanism of either Pt either Co.

Although it is clear that the degradation rate of both metals is highly dependent on the hold time at UPL, before a more comprehensive explanation of this conclusion, let us focus now on the varying potential window. In accordance with the assumptions from the results obtained using the HT-DE methodology (Figure 3), the signals for both Pt and Co dissolution decrease substantially with the narrowing potential window from 0.6–0.95 V_RHE_ (Figure 4a, c,e) to 0.7–0.85 V_RHE_ (Figure 4b,d,f) regardless of the applied hold time at each potential. This has also been reported by previous studies,^43,45,48,66,68,69^ but what is specific here is that both LPL and UPL are nevertheless limited to a realistic operational voltage while positively affecting the stability of both metals. What is also important to emphasize here is that dissolution during cycling when limiting both potential limits (LPL as well as UPL) is, however, not completely suppressed but only significantly reduced, i.e., minimized.

**Figure 6:**
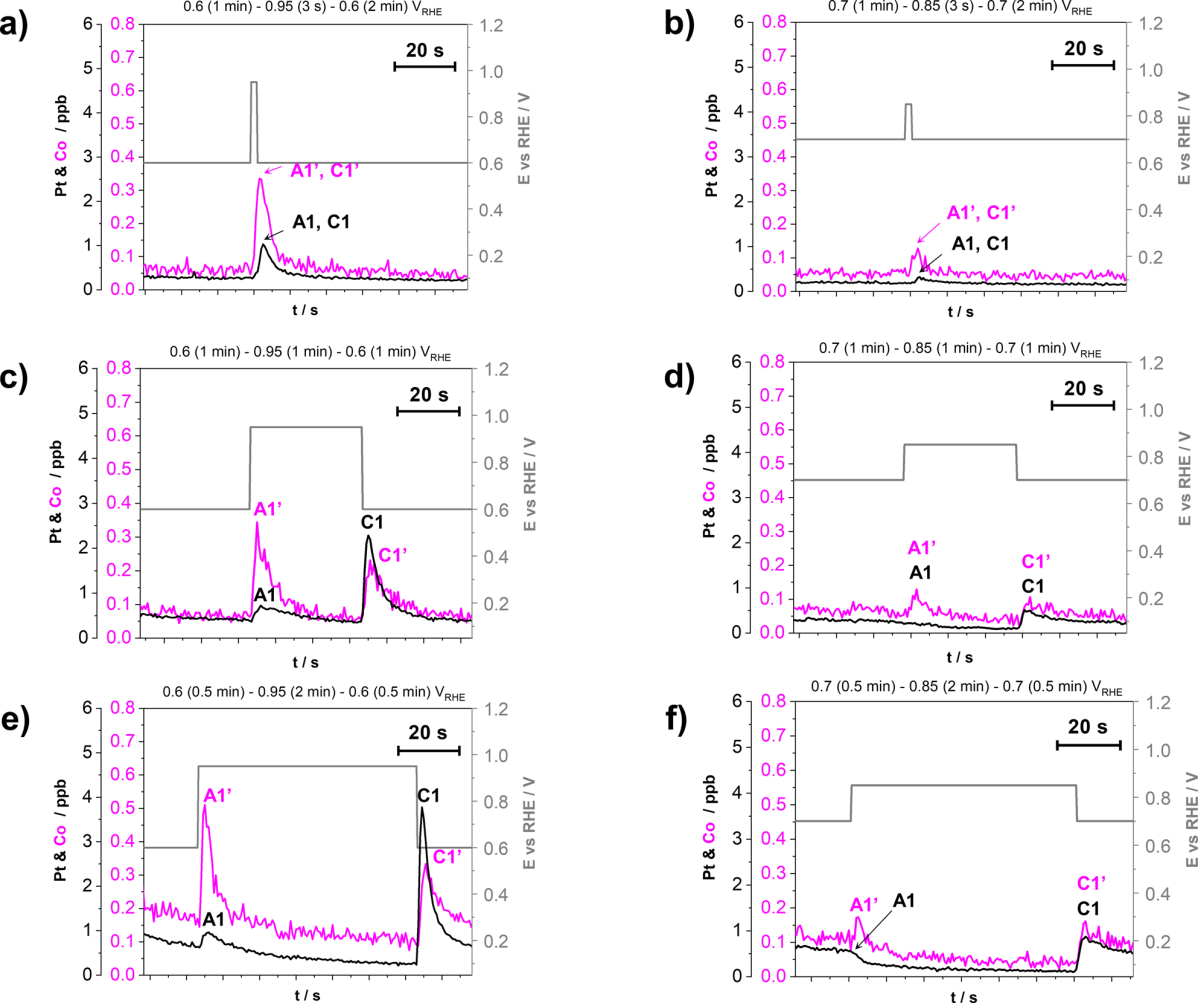
Effect of potential window (0.6–0.95 and 0.7–0.85 V_RHE_) and different hold times at both LPL and UPL on the metal dissolution mechanism (Pt and Co) during trapezoidal wave cycling between LPL and UPL (0.7 and 0.3 V s^–1^, respectively, 10 cycles in total) demonstrated using the EFC-ICP-MS setup in the flow of 0.1 M HClO_4_. Three different protocols per cycle were used: (a, b) LPL (1 min)–UPL (3 s)–LPL (2 min), (c, d) LPL (1 min)–UPL (1 min)–LPL (1 min), and (e, f) LPL (0.5 min)–UPL (2 min)–LPL (0.5 min). For better resolution, close-up metal dissolution profiles of one cycle only (fourth cycle, where the dissolution rate approached steady-state value and there is no effect of a fresh electrocatalyst film) are presented. Each metal has its own *Y* axis to better compare the profiles despite the detected concentration differences. A1 and A1’ represent peaks corresponding to anodic dissolution, whereas C1 and C1’ represent peaks corresponding to the cathodic dissolution of Pt and Co, respectively. The gray lines represent the cycles between LPL and UPL. Supplementary experiment during cycling between LPL (3 s) and UPL (3 s) is provided in SI Figure S10. Whole metal dissolution profiles of a total of 10 cycles for each protocol for both experimental ReCatalyst Pt-Co/C and benchmark from Umicore (Elyst Pt30 0690) are also available in SI Figures S11 and Figure S12, respectively.

Figure 6, on the other hand, focuses on only a single (the same) cycle of each ADT protocol, obtaining the close-up of the metal dissolution profiles and providing an improved understanding of the above-described results (Figures 4 and 5) on the effect of the hold time at UPL as well as the potential window effect. As already described in the Experimental Section, because of the limitations of the peak resolution EFC-ICP-MS at such narrow potential windows, the hold time for ADTs proposed by DoE (3 s for both LPL and UPL) has been extended instead (1 min). Nevertheless, in the first protocol, the hold time at UPL has been kept at 3 s (see Scheme 1a from the Experimental Section, and Figure 6a,b). At such a short hold at UPL, one is unable to sufficiently distinguish between the anodic and cathodic dissolution of Pt and Co (Figure 6a,b), and only a single notable peak for both metals is observed. What is expected, but not yet widely documented for the operational voltages, is that the cathodic transient dissolution of Pt and consequently also Co related to the oxide place-exchange is more damaging than anodic transient dissolution.^10,31,43,46−48,66^ Nevertheless, using the first protocol, we are unable to deconvolute between both contributions precisely. To improve the resolution between both the anodic as well as cathodic metal dissolution peaks, the UPL hold time has been extended to 1 min (second protocol: see Scheme 1b from the Experimental Section, and Figure 6c,d) and 2 min (third protocol: see Scheme 1c from the Experimental Section, and Figure 6e,f) for both potential windows (0.6–0.95 and 0.7–0.85 V_RHE_). For both 1 and 2 min UPL hold times, two peaks corresponding to Pt and Co dissolution are revealed. The first pair of peaks (A1 and A1’) corresponds to the anodic transient dissolution of Pt and Co, respectively, which occurs directly at the change in potential from LPL to UPL. The second pair of peaks (C1 and C1’), on the other hand, corresponds to the well-known—but not yet well documented in the operational window^99^—cathodic transient dissolution resulting from the oxide place-exchange mechanism,^10,46,47^ which is initiated directly at the change in potential from UPL to LPL. Briefly, as we reach the UPL, i.e., during the anodic process, the oxidation of the surface occurs, when only a small part of Pt (and consequently Co) dissolves faster than they could be passivated by the Pt oxide formation (anodic dissolution, denoted as A1 and A1’, respectively; see Figure 6c–f), whereas the rest are passivated by the formation of Pt oxide via adsorption of additional oxygen species and are consequently protected against further dissolution. Afterward, part of this Pt oxide also starts to penetrate the topmost surface layers of the Pt-rich overlayer. Upon the change in potential from UPL to LPL, the reduction of the surface occurs, and at least part of this Pt oxide is reduced, which results in the formation of a large number of highly unstable low-coordinated Pt atoms, which upon exposure to the acidic environment get immediately dissolved, followed also by the dissolution of Co (cathodic dissolution, denoted as C1 and C1’, respectively; see Figure 6c–f).^10,46−48^ Two additional pieces of information are, however, clear: (i) Prolonging the UPL hold time did not only improve the resolution of the metal dissolution peaks; it also clearly reveals that already at the operational voltages (both 0.6–0.95 V in Figure 6c,e as well as 0.7–0.85 V in Figure 6d,f), cathodic dissolution of Pt as a result of oxide place-exchange mechanism is already the dominant dissolution mechanism. A similar role of LPL and UPL in the dissolution mechanism has been shown in the study of Ahluwalia et al., where they investigated the stability of Pt-Co/C electrocatalyst by online ICP-MS measurements of metal dissolution during trapezoidal wave cycling between 0.4 and 1.0 V_RHE_.^68^ Therefore, the oxide place-exchange mechanism plays an important role not only in a wide potential window^68^ but also in a very narrow operational potential window such as used here (0.6–0.95 and 0.7–0.85 V_RHE_), which was also recently shown by Chattot et al..^99^ (ii) Narrowing the voltage window to only 0.7–0.85 V greatly inhibits both the anodic as well as cathodic dissolution of both Pt and consequently Co and thus could significantly prolong the lifetime of Pt-alloy cathodes in PEMFCs using already existing state-of-the-art Pt-alloy electrocatalysts. In the other words, limiting both potential/voltage limits to 0.7–0.85 V_RHE_ acts advantageously through two next scenarios: (i) (s)lower formation of Pt oxide and consequently also a lower degree of oxide place-exchange and (ii) slower kinetics of the reduction of Pt oxide or perhaps even incomplete reduction of the Pt oxides (in other words, the surface of Pt-based nanoparticles might remain partly passivated).^43,45,48^ In summary, whereas the total amount of dissolved Pt (and Co) is strongly affected by the potential window and the hold time at the UPL, the mechanism of Pt dissolution (and Co dissolution) is independent of the potential window as well as the hold time at UPL. Ultimately, in accordance with previous studies,^31^ it is worth mentioning here that a similar scenario is expected for other Pt-3d transition metal alloys. Namely, although the dissolution mechanism can slightly differ from one 3d transition metal to the other^31^ and additional dissolution peaks can occur (e.g., so-called under potential deposition, typical for Cu),^31,100,101^ its anodic and cathodic dissolution always follows the anodic and cathodic dissolution of Pt regardless of the type of less noble metal (Cu, Fe, Ni, Co).^31^ In other words, we assume that the investigated parameters, i.e., the potential window and the hold time at the UPL, will affect other 3d transition metals in a similar way as presented here.

## 4 Conclusions

In conclusion, this work provides guidelines for stability studies of the state-of-the-art carbon-supported Pt-alloy electrocatalysts in liquid electrolyte half-cells at close-to-real operational conditions. The most widespread accelerated degradation test (ADT) protocol at the membrane electrode assembly (MEA) level is the one proposed by the US Department of Energy (DoE), which includes 30,000 trapezoidal wave cycles between 0.6 and 0.95 V_RHE_ with a 3 s hold at both lower potential/voltage limit (LPL/LVL) and upper potential/voltage limit (UPL/UVL). To overlap the gap between the rotating disk electrode (RDE) and MEA levels of investigation, a similar protocol should be applied at both the RDE and the MEA level. The present work uses a modified and adapted protocol for ADTs in liquid half-cells using high-temperature RDE (in our case, specifically, an in-house designed high-temperature disk electrode; HT-DE), which balances the throughput (making it a 20 hour experiment) together with obtaining adequate information on the Pt-based electrocatalyst durability. Namely, 10,000 trapezoidal wave cycles between 0.6 and 0.95 V_RHE_ (0.7 V s^–1^) in 0.1 M HClO_4_ with potential holds of 3 s at both the UPL as well as LPL are proposed to obtain sufficient information on the loss of electrochemically active surface area (ECSA), specific activity (SA), as well as mass activity (MA). Additionally, using the same methodology, it has been shown that the loss of ECSA, SA, and MA decrease dramatically when the applied ADT potential/voltage window is narrowed to only 0.7–0.85 V instead of the typical 0.6–0.95 V.

Furthermore, using the electrochemical flow cell coupled to an inductively coupled plasma mass spectrometry (EFC-ICP-MS) methodology, a deeper understanding of the mechanisms of such ADT degradations has been obtained. It has been shown that the cathodic transient dissolution of Pt is the dominant degradation mechanism already at a potential/voltage window as narrow as 0.7–0.85 V. Thus, although oxide place-exchange has been already widely acknowledged in the PEMFC scientific community as the more damaging degradation mechanism for Pt-based cathodes, our work presents clear evidence that this is also the case in the operational window of the PEMFC already at ambient temperature conditions. In other words, the mechanism of Pt dissolution seems to be rather independent of the potential/voltage window, with cathodic transient dissolution always dominating the dissolution caused by anodic oxidation.

Lastly, the present work also provides evidence that regardless of the used operational potential/voltage window, anodic transient dissolution is not significantly impacted by the increase in the length of the potential hold at the LPL (from 3 s to up to 2 min), whereas the cathodic dissolution as a result of increasing effects of the oxide place-exchange from prolonged potential holds at the UPL is (also from 3 s to up to 2 min). Finally, anodic and cathodic Co dissolution follows anodic and cathodic Pt dissolution, respectively, regardless of the applied potential window (0.6–0.95 or 0.7–0.85 V_RHE_) and duration of hold at UPL (3 s, 1 min, or 2 min).

In relation to this, the work serves as a good motivation for both the entire PEMFC community and the industry to consider Pt-alloy electrocatalysts and Pt-alloy cathodes completely differently from pure-Pt systems, which might be the case when considering not only the durability behavior, which is in the end limited by the durability of Pt itself, but also the way we produce, postprocess, and apply this next generation of materials in PEMFCs. Whereas intrinsic improvements in properties are of extreme importance, much can already be achieved by understanding the strengths and the limitations of Pt-alloys from the electrocatalyst production to the design of the MEAs but perhaps even more importantly the PEMFC stacks with imposed system-level limitations of, for instance, operational voltages. To reach the highly ambitious heavy-duty targets of 30,000 h of operations, there seems to be a clear benefit of achieving peak power densities in MEAs at voltages higher than 0.6 V to inhibit the cathodic metal dissolution as a consequence of the oxide place-exchange while at the same time limiting also the degree of Pt oxidation by limiting the UVL below 0.95 V as well.

## Supporting Information Available

TEM images of electrocatalysts; particle size distribution of electrocatalysts; metal content in electrocatalysts; comparison of initial electrochemical properties of electrocatalysts (ORR and CO electrooxidation); comparison of electrochemical properties of electrocatalysts before and after various ADTs (ORR and CO electrooxidation); comparison of loss of surface area and kinetic properties of benchmark electrocatalyst after various ADTs (ECSA_CO_, SA, and Co loss); and EFC-ICP-MS measurements of metal dissolution of electrocatalysts (PDF)
