# Supplementary material for: Adjusting the Operational Potential Window as a Tool for Prolonging the Durability of Carbon-Supported Pt-Alloy Nanoparticles as Oxygen Reduction Reaction Electrocatalysts
Source: ACS Catal. 2024 Mar 6;14(6):4303–17. doi: 10.1021/acscatal.3c06251 (PMC10949198; doi:10.1021/acscatal.3c06251)
Supplement: Supplementary file 1 — cs3c06251_si_001.pdf [file cs3c06251_si_001.pdf]

Supporting information: Adjusting the Operational Potential Window as a Tool for Prolonging the Durability of Carbon-Supported Pt-alloy Nanoparticles as Oxygen Reduction Reaction Electrocatalysts

Tina Đukić,[a,b] Léonard Jean Moriau,[a] Iva Klofutar,[a] Martin Šala,[d] Luka Pavko,[e] Francisco Javier González López,[e] Francisco Ruiz-Zepeda,[a] Andraž Pavlišič,[f] Miha Hotko,[a,g] Matija Gatalo,[a,e,*] and Nejc Hodnik [a,g*]

[a] Department of Materials Chemistry, National Institute of Chemistry, Hajdrihova 19, 1001 Ljubljana, Slovenia

[b] Faculty of Chemistry and Chemical Technology, University of Ljubljana, Večna pot 113, 1000 Ljubljana, Slovenia

[d] Department of Analytical Chemistry, National Institute of Chemistry, Hajdrihova 19, 1001 Ljubljana, Slovenia

[e] ReCatalyst d.o.o., Hajdrihova Ulica 19, 1001 Ljubljana, Slovenia

[f] Department of Catalysis and Chemical Reaction Engineering, National Institute of Chemistry, Hajdrihova 19, 1001 Ljubljana, Slovenia

[g] University of Nova Gorica, Vipavska 13, Nova Gorica 5000, Slovenia

* to whom correspondence should be addressed: *matija.gatalo@ki.si*, *nejc.hodnik@ki.si*

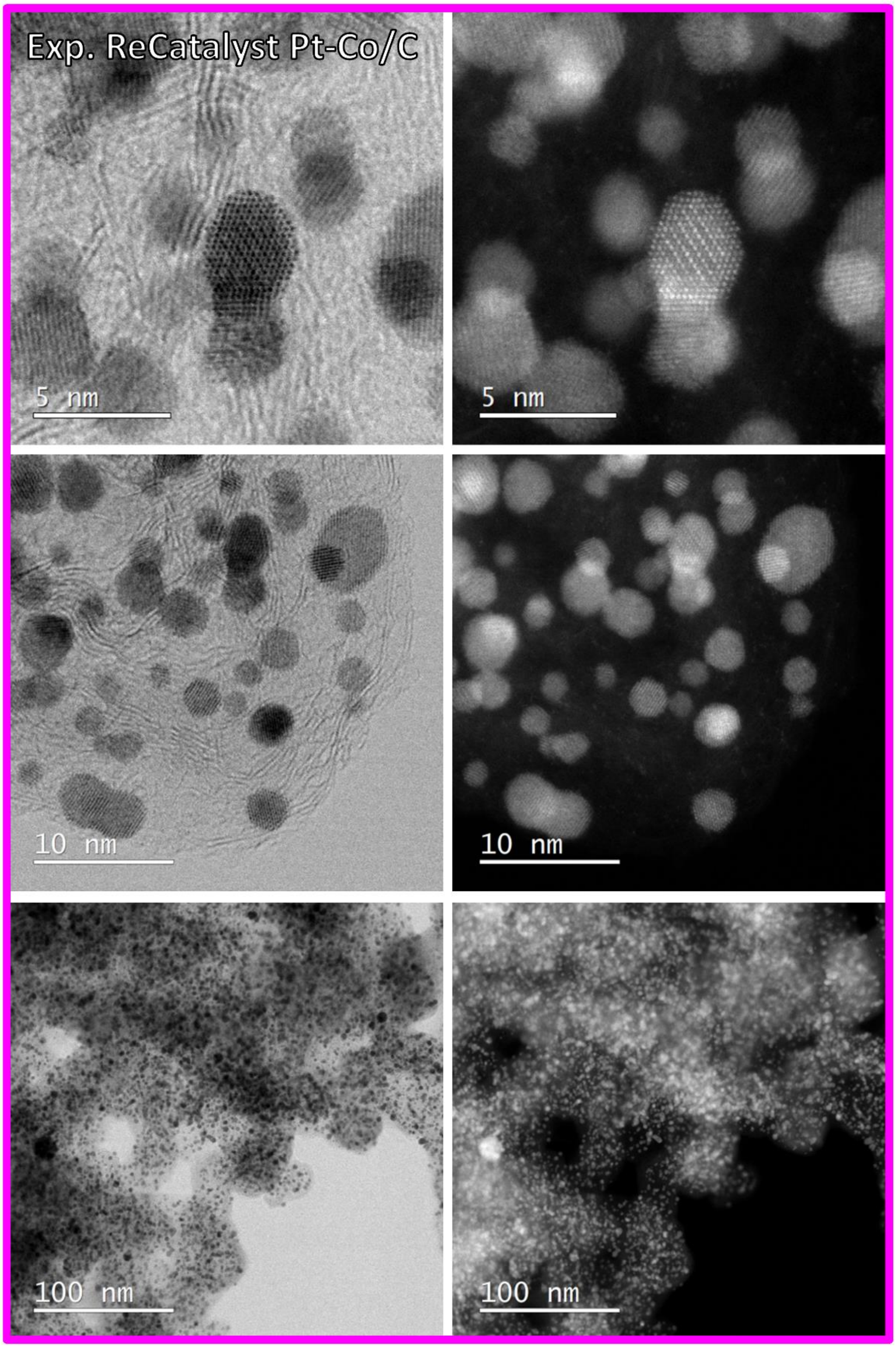

Figure S1. STEM and BF images of the experimental ReCatalyst Pt-Co/C at various magnifications. In all Figures, magenta is used for the data corresponding to the experimental ReCatalyst Pt-Co/C electrocatalyst.

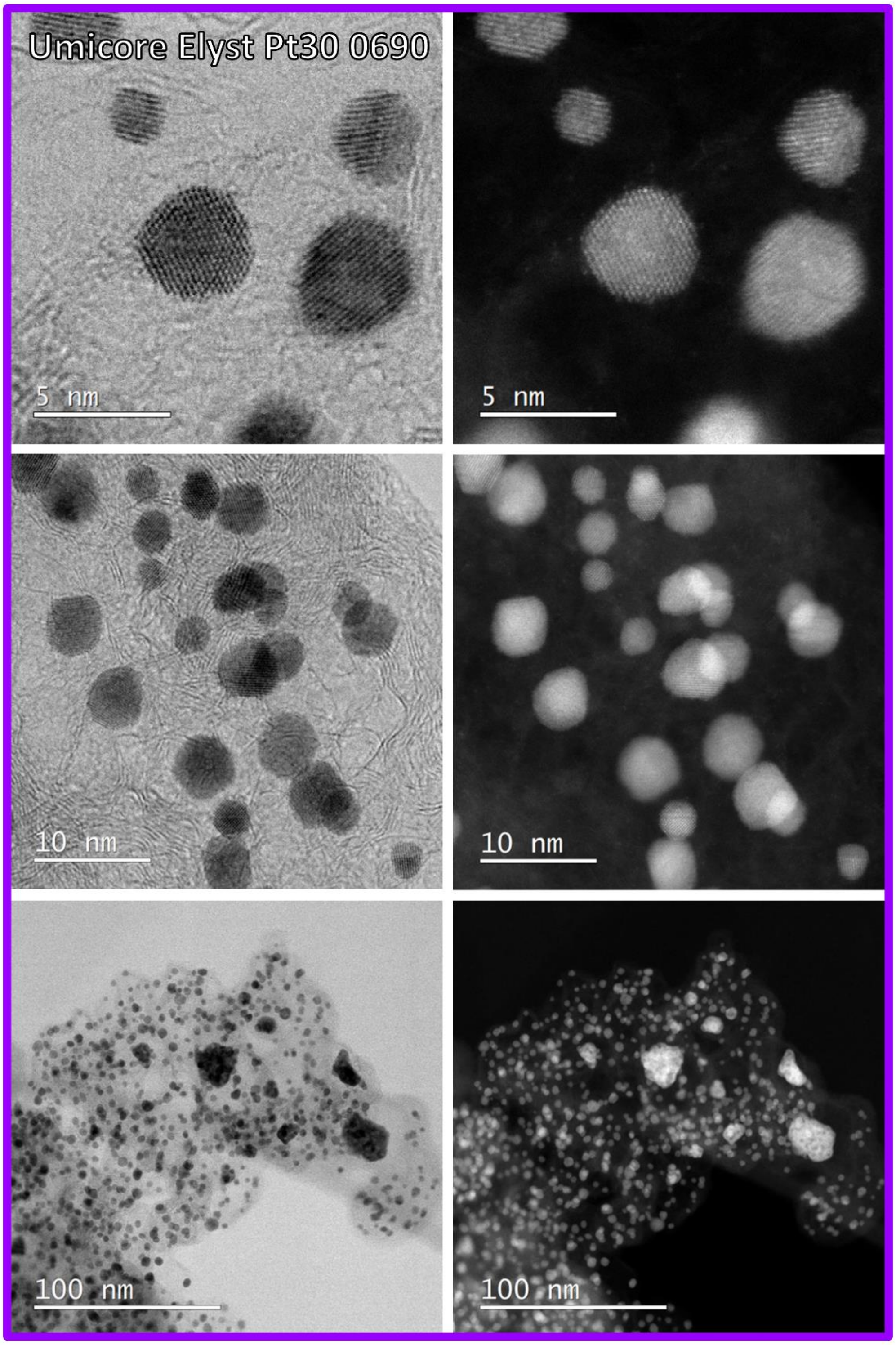

Figure S2. STEM and BF images of the Umicore Elyst Pt30 0690 Pt-Co/C benchmark at various magnifications. In all Figures, violet is used for the data corresponding to the Umicore Elyst Pt30 0690 Pt-Co/C benchmark.

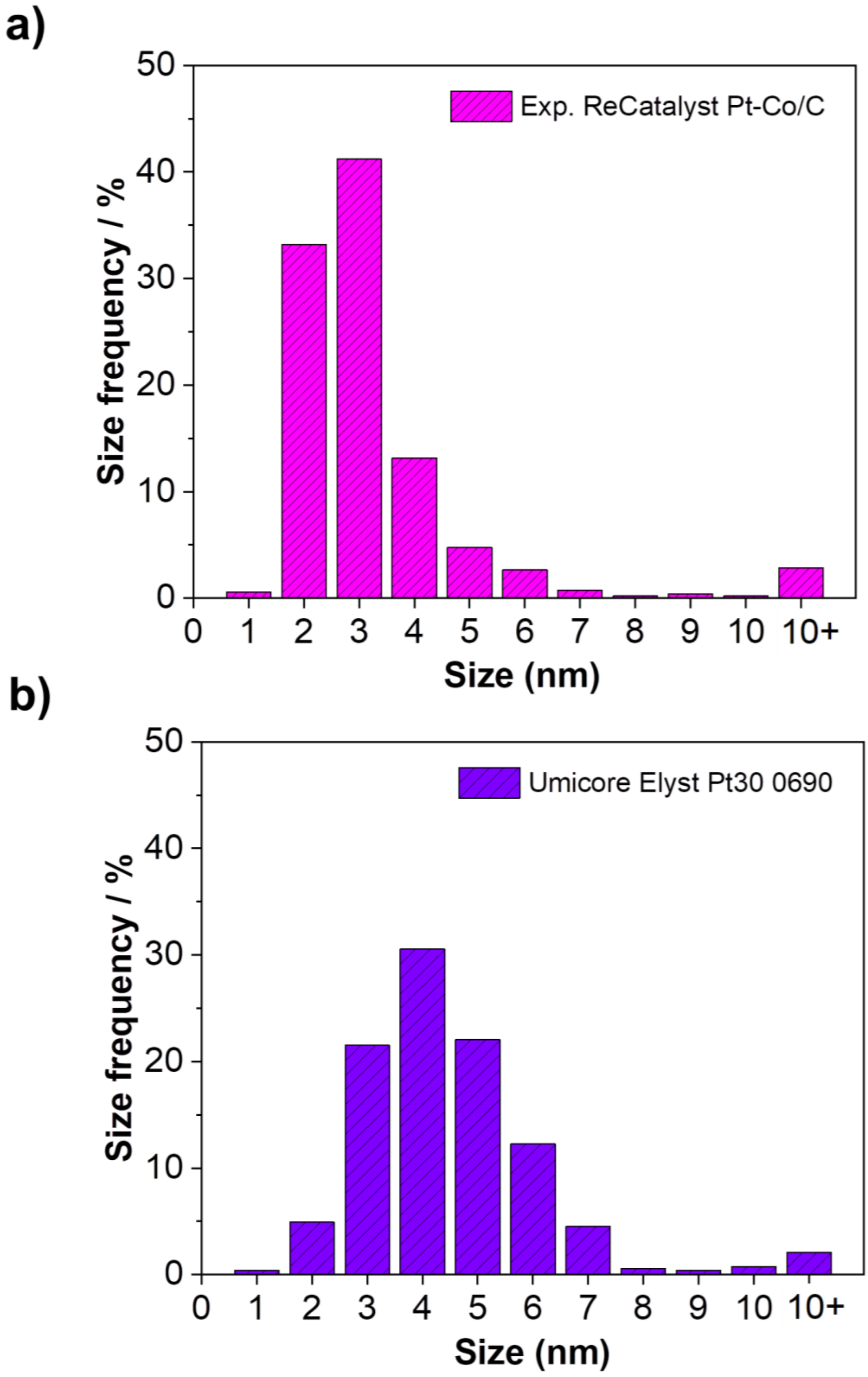

Figure S3. Frequency histogram showing particle size distribution of (a) experimental ReCatalyst Pt-Co/C (counter number of particles = 524) and (b) Umicore Elyst Pt30 0690 (counter number of particles = 530), determined with TEM. In all Figures, magenta is used for the data corresponding to the experimental ReCatalyst electrocatalyst, whereas the data corresponding to the Umicore's benchmark is in violet.

Table S1. Weight percentages of metal content in the samples derived from EDX analysis

**Sample**	**Pt [wt%]**	**Co [wt%]**
Experimental ReCatalyst Pt-Co/C	28.6	1.4
Umicore Elyst Pt30 0690	26	2.7

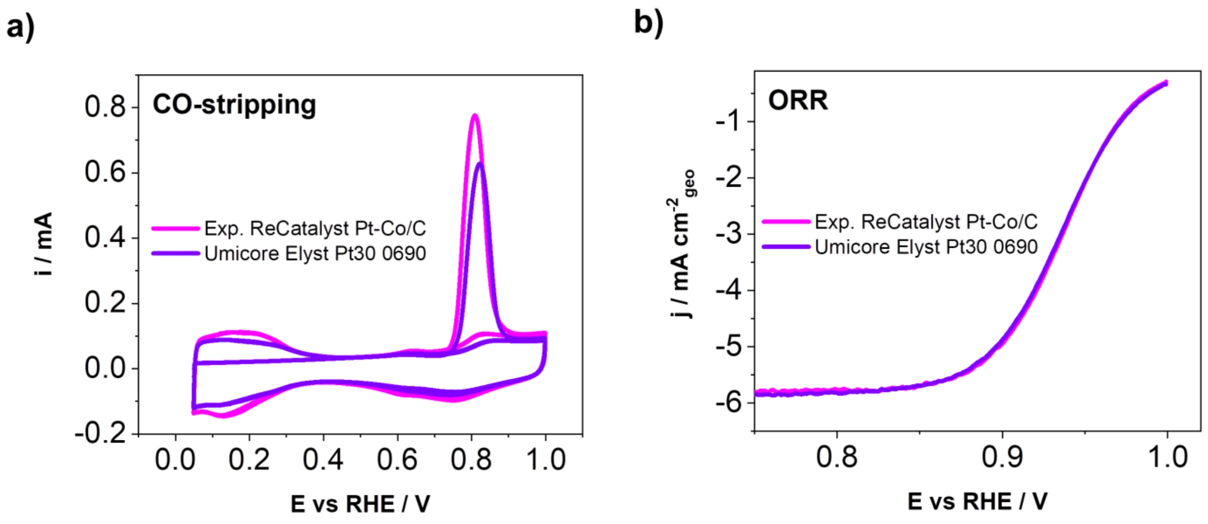

Figure S4. Comparison of **(a)** CO-electrooxidation CVs (0.05–1.0 V_{RHE}, 20 mV s^{-1}, without rotation) as well as **(b)** ORR polarization curves (0.05–1.0 V_{RHE}, 20 mV s^{-1}, 1600 rpm, O_2 saturated, Ohmic resistance compensated) of both the experimental ReCatalyst Pt-Co/C as well as Umicore Elyst Pt30 0690 electrocatalysts, with loading of 20 μg for both electrocatalysts. In all Figures, magenta is used for the data corresponding to the experimental ReCatalyst electrocatalyst, whereas the data corresponding to the Umicore's benchmark is in violet.

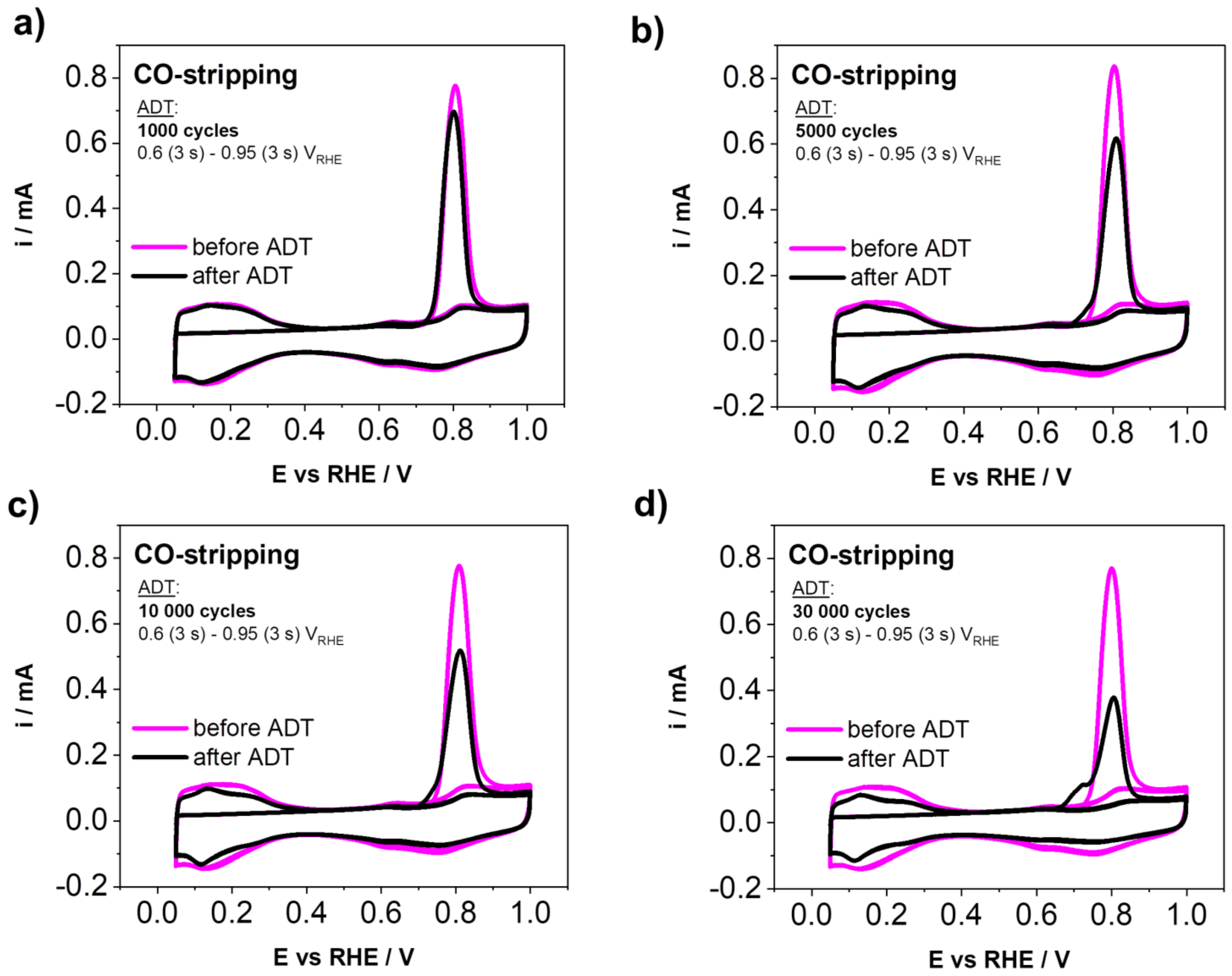

Figure S5. Comparison of CO-electrooxidation CVs as well as the follow-up cycles before and after ADT performed with **(a)** 1000, **(b)** 5000, **(c)** 10,000 and **(d)** 30,000 trapezoidal wave cycles at a constant potential window (0.6 (3 s)–0.95 (3 s) V_{RHE}, 0.7 V s^{-1}, 0.1 M $HClO_4$, 60 °C) for the experimental ReCatalyst Pt-Co/C

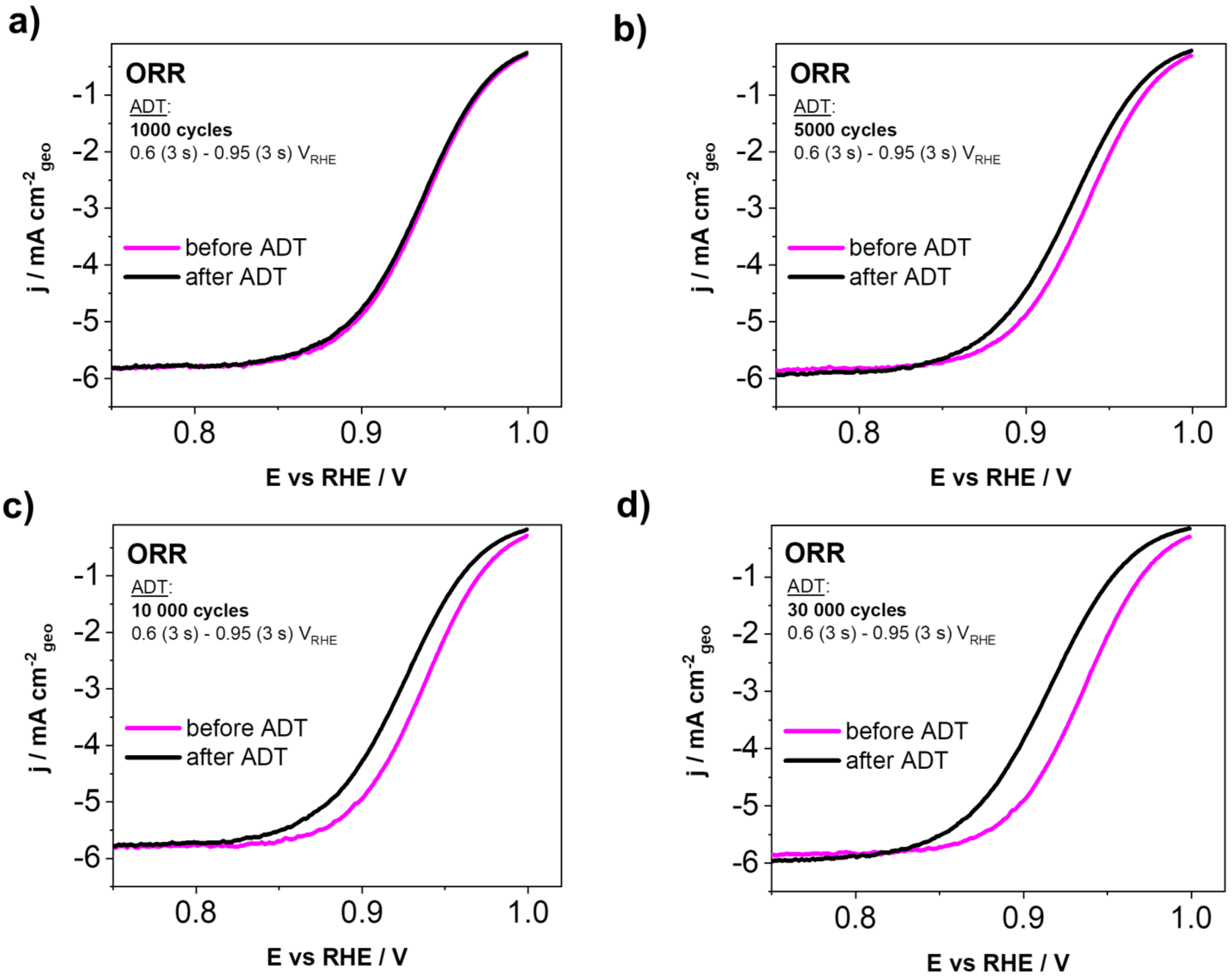

Figure S6. Comparison of ORR polarization curves before and after ADT performed with **(a)** 1000, **(b)** 5000, **(c)** 10,000 and **(d)** 30,000 trapezoidal wave cycles at a constant potential window (0.6 (3 s)–0.95 (3 s) V_{RHE}, 0.7 V s^{-1}, 0.1 M $HClO_4$, 60 °C) for the experimental ReCatalyst Pt-Co/C

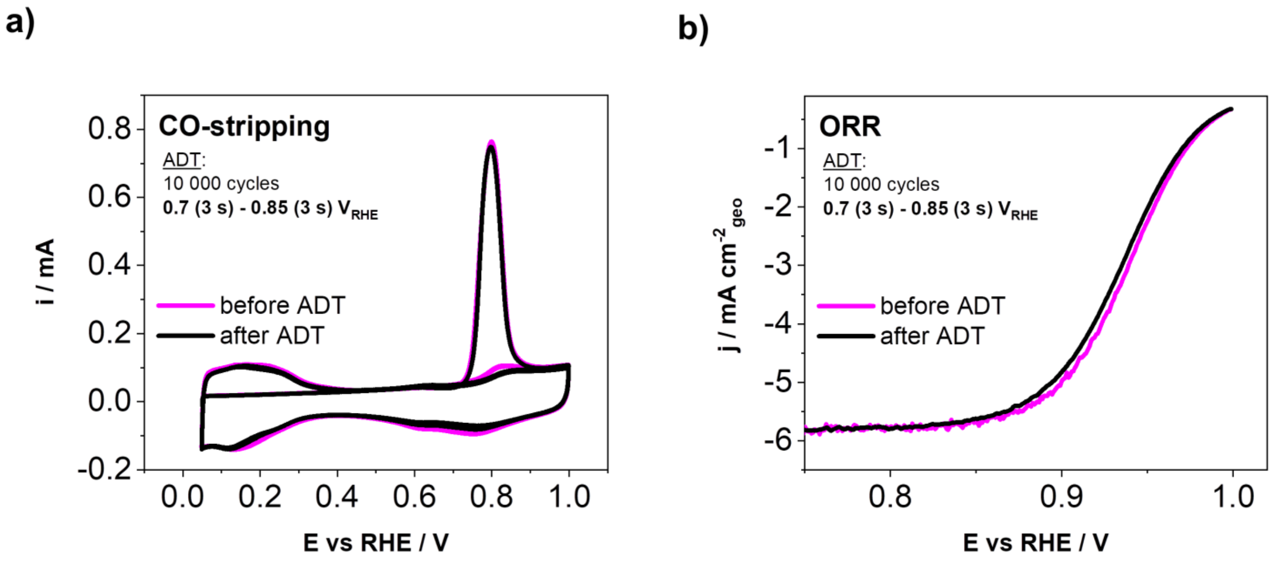

Figure S7. Comparison of **(a)** the CO-electrooxidation CVs as well as the follow-up cycles and **(b)** ORR polarization curves before and after accelerated degradation tests (ADTs) performed by trapezoidal wave cycling (0.7 (3 s)–0.85 (3 s) V_{RHE}; 10,000 cycles, 0.7 V s^{-1}, 0.1 M $HClO_4$, 60 °C) for the experimental ReCatalyst Pt-Co/C

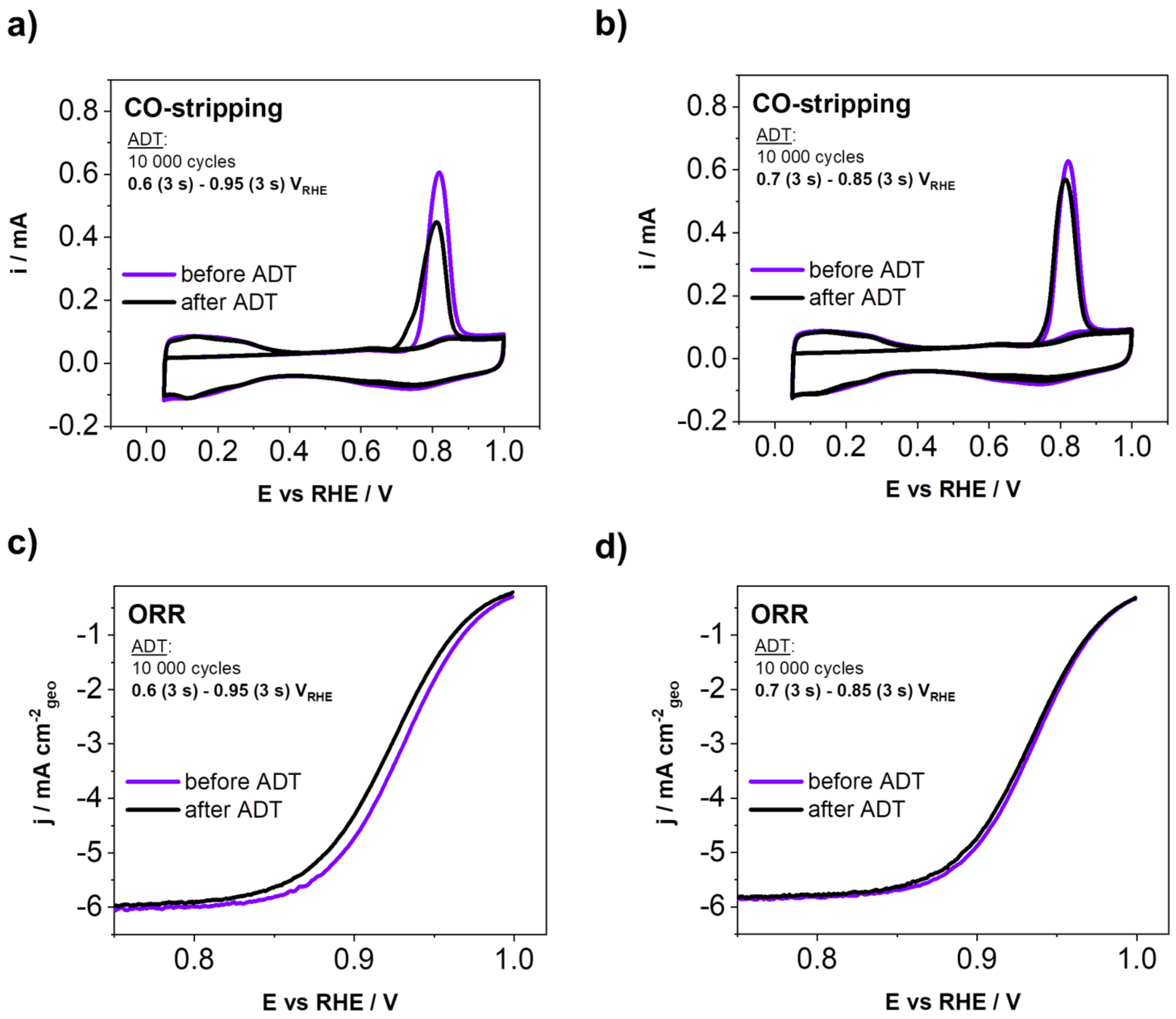

Figure S8. Comparison of **(a–b)** the CO-electrooxidation CVs as well as the follow-up cycles and **(c–d)** ORR polarization curves before and after accelerated degradation tests (ADTs) performed by trapezoidal wave cycling (LPL (3 s)–UPL (3 s); LPL = 0.6, 0.7 V_{RHE} and UPL = 0.85, 0.95 V_{RHE}; 10,000 cycles, 0.7 V s^{-1}, 0.1 M $HClO_4$, 60 °C) for the Umicore Elyst Pt30 0690 Pt-Co/C benchmark

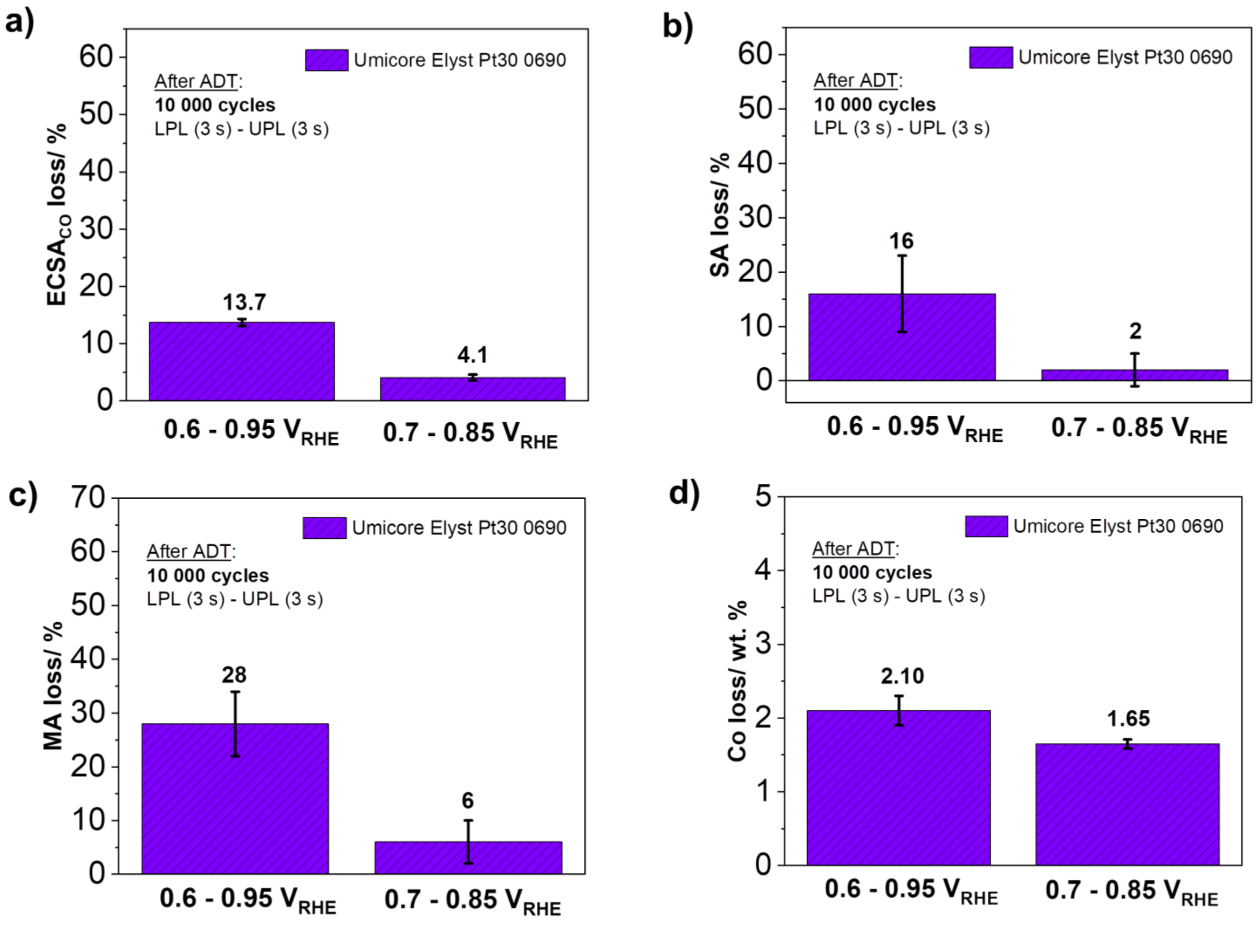

Figure S9. Effect of varying the operational potential window on accelerated degradation tests (ADTs) performed by trapezoidal wave cycling (LPL (3 s)–UPL (3 s); LPL = 0.6, 0.7 V_{RHE} and UPL = 0.85, 0.95 V_{RHE}; 10,000 cycles, 0.7 V s^{-1}, 0.1 M $HClO_4$, 60 °C): **(a)** $ECSA_{CO}$ loss, **(b)** SA loss, **(c)** MA loss, and **(d)** Co loss of the Umicore Elyst Pt30 0690 Pt-Co/C benchmark

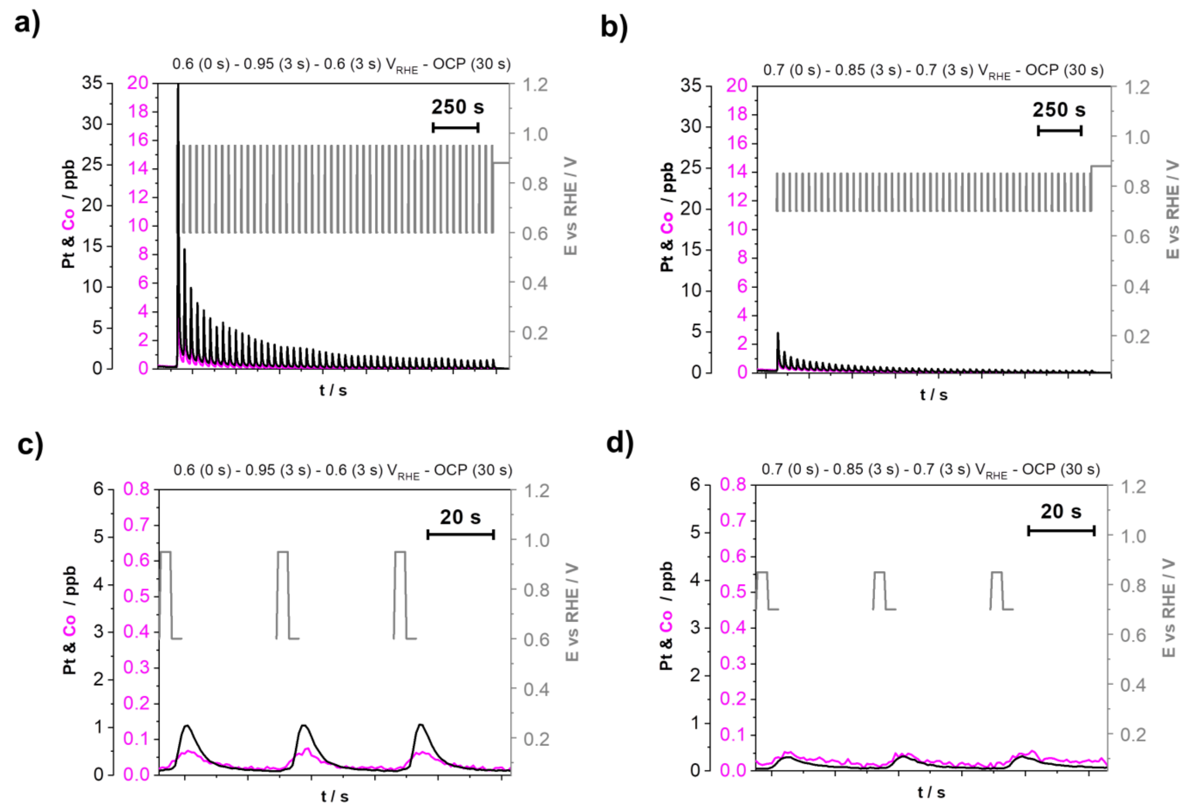

Figure S10. Effect of potential window (0.6–0.95 V_{RHE} and 0.7–0.85 V_{RHE}) on the metal dissolution (Pt and Co) of the experimental ReCatalyst Pt-Co/C electrocatalyst during trapezoidal wave cycling at LPL (3 s)–UPL (3 s) (0.7 V s^{-1}, 50 cycles in total), demonstrated using the EFC-ICP-MS setup in the flow of 0.1 M $HClO_4$. **(a–b)** All 50 cycles, **(c–d)** Close-up metal dissolution profiles of three cycles. Each metal has its own *Y* axis to better compare the profiles despite the detected concentration differences. The gray lines represent the cycles between LPL and UPL.

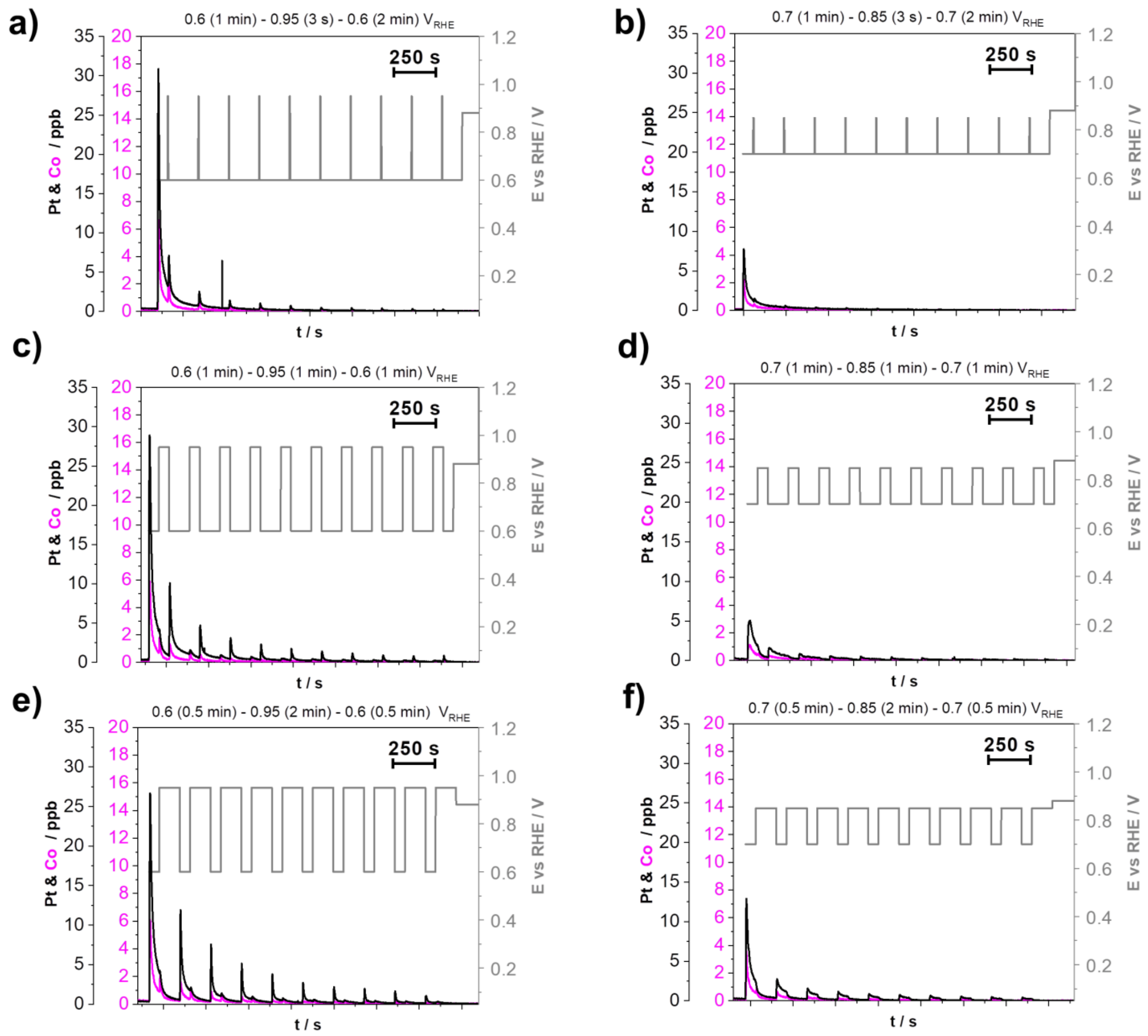

Figure S11. Effect of potential window (0.6–0.95 V_{RHE} and 0.7–0.85 V_{RHE}) and different hold time at both LPL and UPL on the metal dissolution (Pt and Co) of the experimental ReCatalyst Pt-Co/C electrocatalyst during trapezoidal wave cycling between LPL and UPL (0.7 V s^{-1}, 10 cycles in total), demonstrated using the EFC-ICP-MS setup in the flow of 0.1 M $HClO_4$. All 10 cycles are presented. Three different protocols per cycle were used: **(a–b)** LPL (1 min)–UPL (3 s)–LPL (2 min) V_{RHE}, **(c–d)** LPL (1 min)–UPL (1 min)–LPL (1 min) V_{RHE}, **(e–f)** LPL (0.5 min)–UPL (2 min)–LPL (0.5 min) V_{RHE}. Each metal has its own *Y* axis to better compare the profiles despite the detected concentration differences. The gray lines represent the cycles between LPL and UPL.

Table S2. Summarized data on the amount of dissolved Pt and Co from experimental ReCatalyst Pt-Co/C electrocatalyst, obtained by integrating corresponding peaks from Figure S11. Amount of dissolved Pt and Co was obtained by multiplication of the area under dissolution curve to the electrolyte flow rate, which was constant for all experiments (400 μL min^{-1}).

<table>
<tr><th colspan="2"></th><th colspan="3">Pt</th><th colspan="3">Co</th></tr>
<tr><th colspan="2">Protocol</th><th>Area under dissolution curve for 10 cycles [ng s mL^{-1}]</th><th>Dissolved in 10 cycles [ng]</th><th>Dissolved in 10 cycles [%]</th><th>Area under dissolution curve for 10 cycles [ng s mL^{-1}]</th><th>Dissolved in 10 cycles [ng]</th><th>Dissolved in 10 cycles [%]</th></tr>
<tr><td rowspan="3">0.6–0.95 V_{RHE}</td><td>LPL (1 min)–UPL (3 s)–LPL (2 min)</td><td>727.37</td><td>4.85</td><td>0.085</td><td>175.60</td><td>1.17</td><td>0.418</td></tr>
<tr><td>LPL (1 min)–UPL (1 min)–LPL (1 min)</td><td>1119.55</td><td>7.46</td><td>0.130</td><td>205.71</td><td>1.37</td><td>0.489</td></tr>
<tr><td>LPL (0.5 min)–UPL (2 min)–LPL (0.5 min)</td><td>1316.36</td><td>8.78</td><td>0.153</td><td>245.13</td><td>1.63</td><td>0.582</td></tr>
<tr><td rowspan="2">0.7–0.85 V_{RHE}</td><td>LPL (1 min)–UPL (3 s)–LPL (2 min)</td><td>250.97</td><td>1.67</td><td>0.029</td><td>60.54</td><td>0.40</td><td>0.143</td></tr>
<tr><td>LPL (1 min)–UPL (1 min)–LPL (1min)</td><td>442.08</td><td>2.95</td><td>0.052</td><td>80.35</td><td>0.54</td><td>0.193</td></tr>
</table>

	LPL (0.5 min)–UPL (2 min)–LPL (0.5 min)	626.19	4.17	0.073	133.11	0.89	0.318

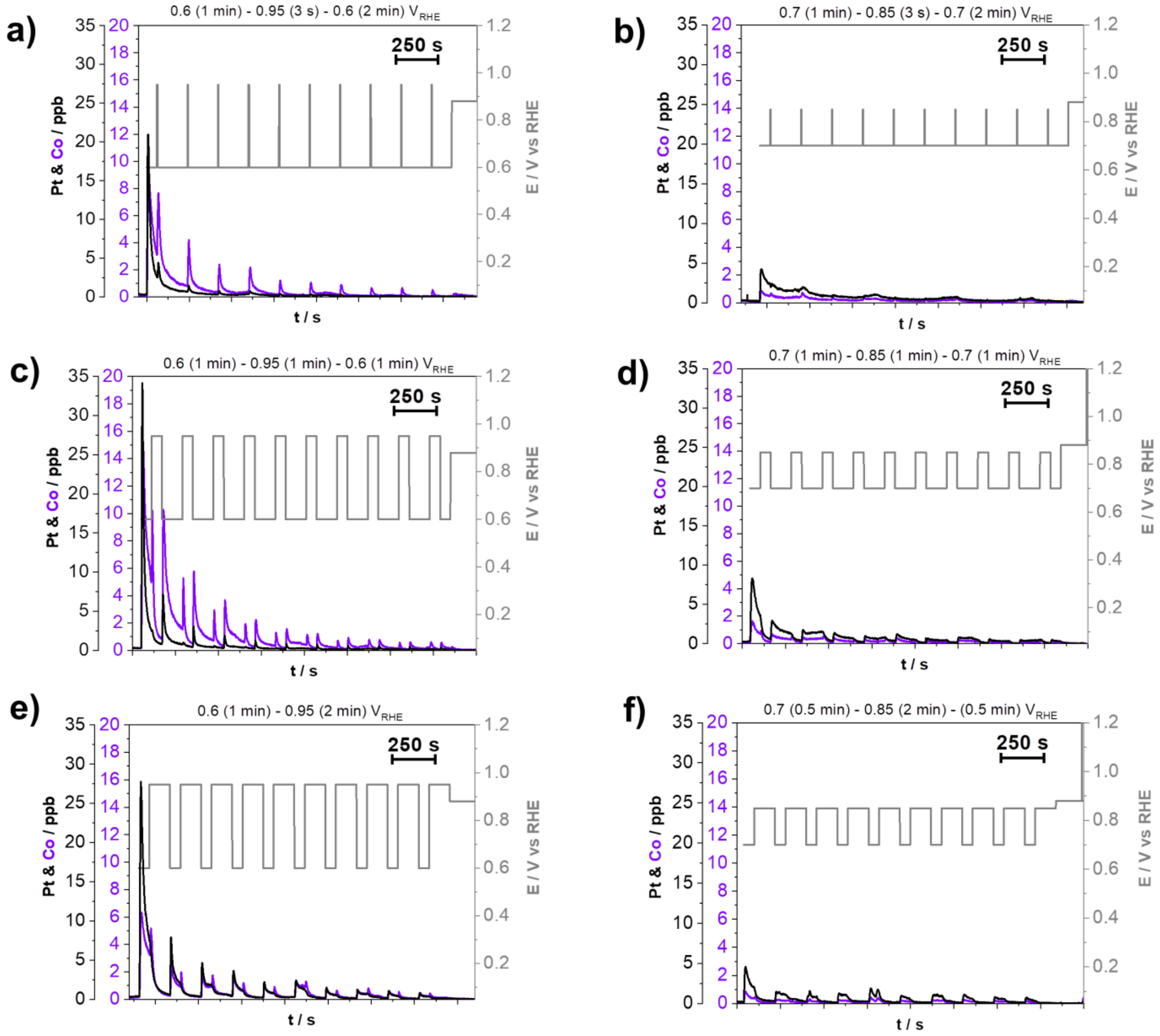

Figure S12. Effect of potential window (0.6–0.95 V_{RHE} and 0.7–0.85 V_{RHE}) and different hold time at both LPL and UPL on the metal dissolution (Pt and Co) of the Umicore Elyst Pt30 0690 Pt-Co/C benchmark during trapezoidal wave cycling between LPL and UPL (0.7 V s^{-1}, 10 cycles in total), demonstrated using the EFC-ICP-MS setup in the flow of 0.1 M $HClO_4$. All 10 cycles are presented. Three different protocols per cycle were used: **(a–b)** LPL (1 min)–UPL (3 s)–LPL (2 min) V_{RHE}, **(c–d)** LPL (1 min)–UPL (1 min)–LPL (1 min) V_{RHE}, **(e–f)** LPL (0.5 min)–UPL (2 min)–LPL (0.5 min) V_{RHE}. Each metal has its own *Y* axis to better compare the profiles despite the detected concentration differences. The gray lines represent the cycles between LPL and UPL.
